# A Mobile Health Intervention Supporting Heart Failure Patients and Their Informal Caregivers: A Randomized Comparative Effectiveness Trial

**DOI:** 10.2196/jmir.4550

**Published:** 2015-06-10

**Authors:** John D Piette, Dana Striplin, Nicolle Marinec, Jenny Chen, Ranak B Trivedi, David C Aron, Lawrence Fisher, James E Aikens

**Affiliations:** ^1^ Center for Clinical Management Research and Center for Managing Chronic Disease VA Ann Arbor Healthcare System and University of Michigan School of Public Health Ann Arbor, MI United States; ^2^ Center for Innovation to Implementation and Department of Psychiatry and Behavioral Sciences VA Palo Alto Health Care System and Stanford University Palo Alto, CA United States; ^3^ Louis Stokes VA Cleveland Medical Center and Department of Medicine, Case Western Reserve University Cleveland, OH United States; ^4^ Department of Family and Community Medicine University of California-San Francisco San Francisco, CA United States; ^5^ Department of Family Medicine University of Michigan Ann Arbor, MI United States

**Keywords:** telehealth, mobile health, heart failure, disease management, self-management

## Abstract

**Background:**

Mobile health (mHealth) interventions may improve heart failure (HF) self-care, but standard models do not address informal caregivers’ needs for information about the patient’s status or how the caregiver can help.

**Objective:**

We evaluated mHealth support for caregivers of HF patients over and above the impact of a standard mHealth approach.

**Methods:**

We identified 331 HF patients from Department of Veterans Affairs outpatient clinics. All patients identified a “CarePartner” outside their household. Patients randomized to “standard mHealth” (n=165) received 12 months of weekly interactive voice response (IVR) calls including questions about their health and self-management. Based on patients’ responses, they received tailored self-management advice, and their clinical team received structured fax alerts regarding serious health concerns. Patients randomized to “mHealth+CP” (n=166) received an identical intervention, but with automated emails sent to their CarePartner after each IVR call, including feedback about the patient’s status and suggestions for how the CarePartner could support disease care. Self-care and symptoms were measured via 6- and 12-month telephone surveys with a research associate. Self-care and symptom data also were collected through the weekly IVR assessments.

**Results:**

Participants were on average 67.8 years of age, 99% were male (329/331), 77% where white (255/331), and 59% were married (195/331). During 15,709 call-weeks of attempted IVR assessments, patients completed 90% of their calls with no difference in completion rates between arms. At both endpoints, composite quality of life scores were similar across arms. However, more mHealth+CP patients reported taking medications as prescribed at 6 months (8.8% more, 95% CI 1.2-16.5, *P*=.02) and 12 months (13.8% more, CI 3.7-23.8, *P*<.01), and 10.2% more mHealth+CP patients reported talking with their CarePartner at least twice per week at the 6-month follow-up (*P*=.048). mHealth+CP patients were less likely to report negative emotions during those interactions at both endpoints (both *P*<.05), were consistently more likely to report taking medications as prescribed during weekly IVR assessments, and also were less likely to report breathing problems or weight gains (all *P*<.05). Among patients with more depressive symptoms at enrollment, those randomized to mHealth+CP were more likely than standard mHealth patients to report excellent or very good general health during weekly IVR calls.

**Conclusions:**

Compared to a relatively intensive model of IVR monitoring, self-management assistance, and clinician alerts, a model including automated feedback to an informal caregiver outside the household improved HF patients’ medication adherence and caregiver communication. mHealth+CP may also decrease patients’ risk of HF exacerbations related to shortness of breath and sudden weight gains. mHealth+CP may improve quality of life among patients with greater depressive symptoms. Weekly health and self-care monitoring via mHealth tools may identify intervention effects in mHealth trials that go undetected using typical, infrequent retrospective surveys.

**Trial Registration:**

ClinicalTrials.gov NCT00555360; https://clinicaltrials.gov/ct2/show/NCT00555360 (Archived by WebCite at http://www.webcitation.org/6Z4Tsk78B).

## Introduction

Chronic heart failure (HF) is associated with reduced quality of life, preventable hospitalizations, and early mortality [1,2]. For effective disease management, patients must systematically monitor symptoms, including shortness of breath, weight gain, and edema, and follow strict self-care practices including limiting salt and fluid intake, and taking medications as prescribed [3,4]. Because HF management is challenging, patients frequently experience life-threatening exacerbations that are responsible for $40 billion in US health care costs each year [5]. Telephone care management can improve HF patients’ prognosis [6-10]. However, telephone follow-up is inadequately reimbursed and competes with in-person care for clinicians’ time [11].

A number of recently completed clinical trials and evidence syntheses have shown that mobile health (mHealth) interventions can improve self-care behaviors and physiologic risk factors for poor outcomes of cardiovascular disease, including heart failure [12-16]. For example, risk factor management using interactive voice response (IVR) calls can improve dietary behaviors and blood pressure control among hypertension patients in the United States and Latin America [17-19], and remote monitoring coupled with self-management assistance has been shown to improve outcomes of cardiovascular disease in a number of countries [20-25]. Despite these encouraging findings, not all trials of HF self-management support via mHealth tools have shown positive outcomes [26,27]. Without substantial restructuring of financial incentives for health care organizations and systems to follow up on identified problems, increased monitoring may be insufficient to fill the gap between what HF patients need and what health systems can provide [28,29].

One potential solution to bridging the gap between the promise and the practice of mHealth self-management assistance may be to expand the reach of interventions so that they support not only patients but also their informal caregivers. Informal caregivers often help chronically ill patients follow self-management recommendations by providing support that is unavailable through professional care management [30-33]. However, in-home caregivers are often elderly, ill, and overwhelmed [34,35]. Most in-home caregivers lack the training and resources needed to systematically monitor HF patients and provide self-management assistance. Moreover, chronically ill patients increasingly have caregivers outside of the household, making health and self-care monitoring much more difficult [36,37].

The CarePartner program was developed through a series of Veterans Affairs (VA) and non-VA pilot and feasibility studies to address these challenges by enabling structured support by informal caregivers (CarePartners) who reside outside the patient’s home. Through this program, patients receive regular monitoring and tailored self-management education via IVR calls with feedback to their clinician. While evidence suggests that between-visit mHealth assistance could be effective in improving HF self-care and outcomes, it remains unclear whether feedback to CarePartners is helpful over and above the support provided directly to patients and clinicians.

This study reports the results of a randomized comparative effectiveness trial testing the impact of systematic feedback to patients’ CarePartners, compared to patients receiving standard mHealth monitoring and self-management education. Analyses focused on changes in patients’ HF-related quality of life, self-care, and patient-CarePartner communication reported via 6- and 12-month surveys, as well as on patients’ medication adherence and symptoms reported via weekly IVR calls.

CarePartners also completed surveys at 6- and 12-months post enrollment. The primary results of those assessments are presented elsewhere [38]. In brief, CarePartners who experienced significant caregiving strain and depression at baseline experienced significant decreases in those symptoms if randomized to receive systematic feedback about their patient-partner’s health and self-care, and also reported increased engagement in self-management support. In order to provide additional information about the intervention experience from the CarePartners’ perspective, here we briefly describe qualitative feedback from CarePartners at follow-up as well as their unsolicited replies to email reports sent automatically based on patients’ IVR assessment calls.

## Methods

### Recruitment

Patients were recruited from VA Cleveland Medical Center outpatient clinics between June 2009 and January 2012 and were followed for 12 months. To be eligible, patients had to have an HF diagnosis, New York Heart Association classification of II or III, and a documented ejection fraction <40% (see Multimedia Appendix 1). Patients also had to have attended at least one VA outpatient visit within the previous 12 months, have a VA primary care provider, and be able to participate in automated telephone calls in English. Patients needed to nominate an eligible CarePartner, that is, a relative or friend living outside their home. Patients were excluded if they lived in a skilled nursing facility; were prescribed oxygen supplementation; were receiving palliative care; had a life-threating condition such as lung cancer; or had ICD-9 coded diagnoses indicating dementia, bipolar disorder, or schizophrenia.

Potentially eligible patients identified from electronic medical records were sent an invitation letter, followed by a screening and recruitment call. Eligible and interested patients were mailed informed consent forms and were assisted in identifying potential CarePartners using the Norbeck Social Support Questionnaire (NSSQ) [39]. To be eligible, CarePartners had to live outside the patient’s home, speak English, have access to a telephone and email, and report at least monthly contact with the patient. CarePartners provided verbal consent to participate.

### Randomization

After completing baseline surveys, patient-CarePartner dyads were randomized by a research associate to a patient-focused mHealth service (standard mHealth) or a service that included feedback to patients’ CarePartners (mHealth+CP). Pairs were randomized within strata defined by whether the patient had an in-home caregiver. Sealed randomization envelopes were created by the study coordinator in blocks using an online random number generator. It was impossible to blind patients to their random assignment because patients and CarePartners were aware whether the CarePartner received email feedback.

### Standard mHealth Intervention

**Figure 1:**
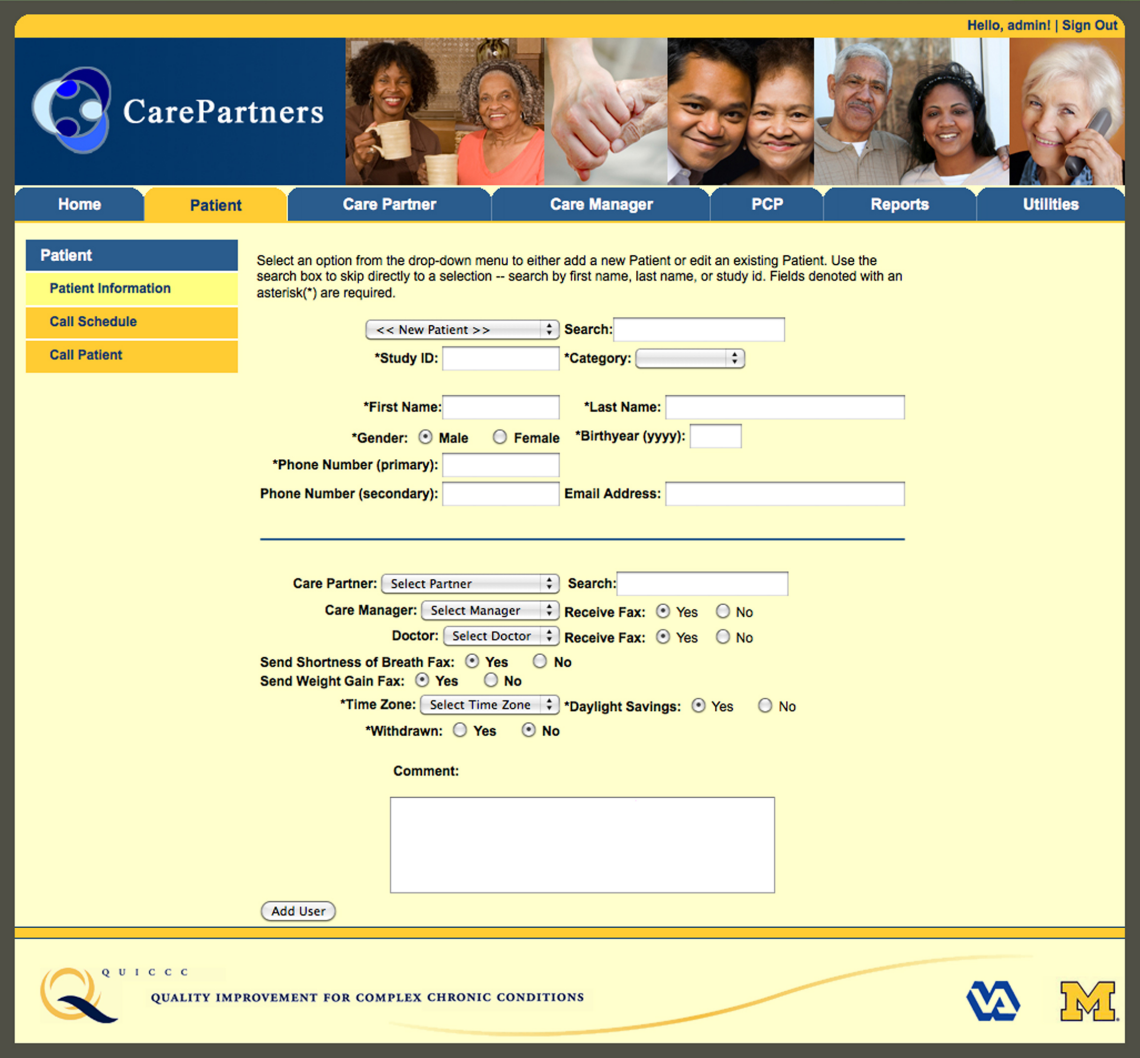
Patient enrollment page.

**Figure 2:**
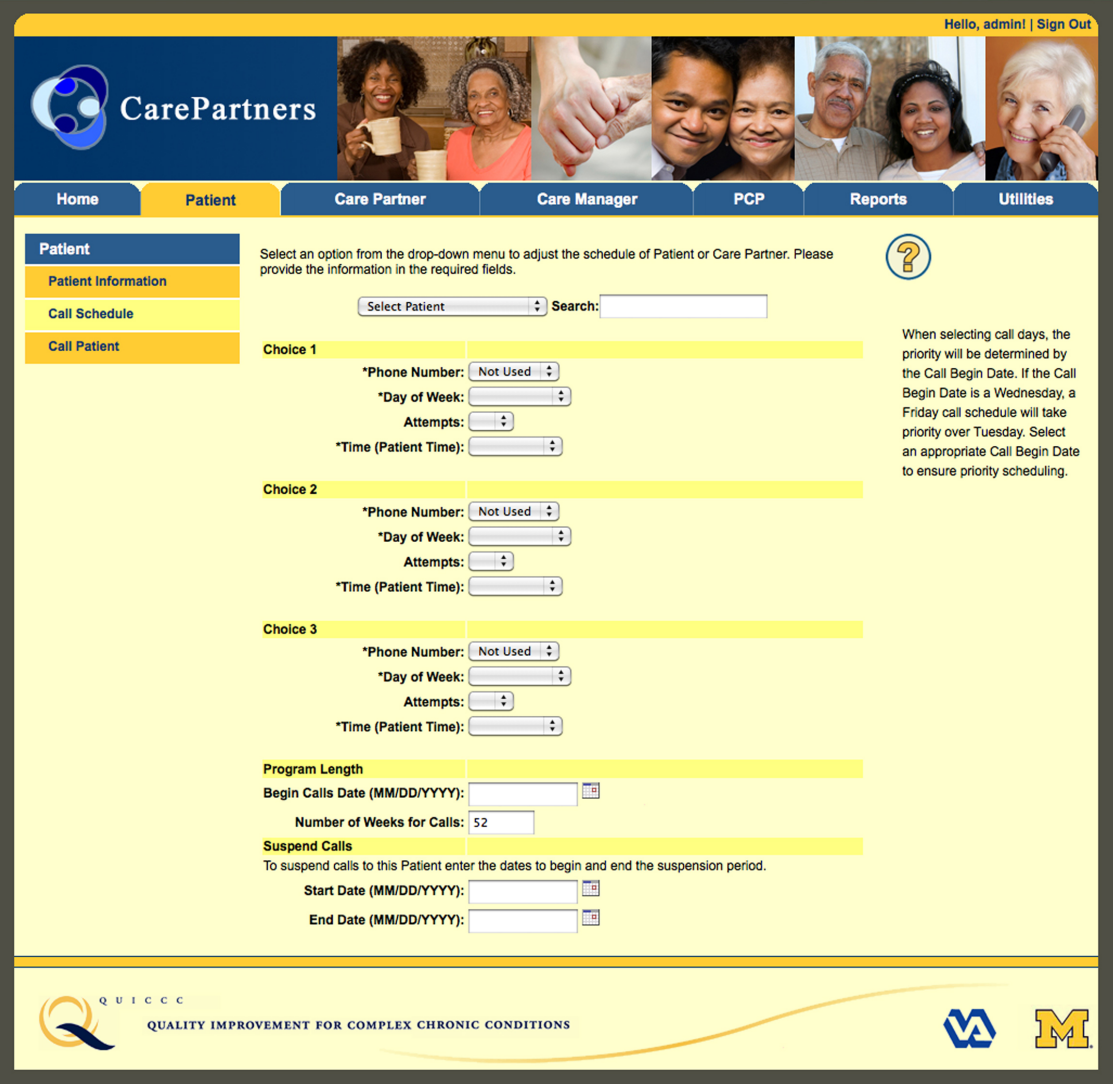
Call scheduling page.

Patients, CarePartners, and in-home caregivers (when present) randomized to standard mHealth were mailed information about HF self-care [37]. Patients received weekly IVR monitoring and self-management support calls for 12 months. Up to nine call attempts per week were made at times the patient indicated were convenient. IVR calls included recorded information and questions that patients answered using their touchtone keypad. The IVR calls were developed by a panel including primary care physicians, cardiologists, nurses, and experts in health behavior change and mHealth. Calls lasted roughly 10 minutes and followed a tree-structured algorithm to ask about overall health, HF symptoms, and self-management behaviors. Patients received pre-recorded information tailored to their reported symptoms and self-care practices. See Figures 1 and 2 for screenshots of the website used for enrollment and call scheduling.

When patients reported an urgent issue via IVR (ie, worsening shortness of breath or a significant weight increase), the system automatically issued a fax notification to their clinician. A significant weight increase was defined as a 5-lb increase over 1 or 2 weeks, a 7-lb increase over 3 weeks, or an average gain of 2 lbs per week since the last automated call if more than 3 weeks had elapsed. Actions taken by clinicians based on the faxes were not tracked.

### mHealth+CP Intervention

The mHealth+CP intervention was based on self-regulation theory, which emphasizes communication of expectations of behavior (“standards”), promotion of motivation to meet standards, and monitoring with feedback regarding the gap between behavior and standards [40,41]. Patients and CarePartners randomized to mHealth+CP received identical intervention elements described above.

mHealth+CP CarePartners were automatically emailed a structured report after each completed IVR call. CarePartner reports were sent to their personal, individual email addresses, which were stored in the system’s secure database at the University of Michigan. Reports described in lay language what patients’ responses meant in terms of risk for HF exacerbations and included suggestions for how CarePartners could support self-management. Email reports referred to the patient using gender-specific pronouns, for example, “Your partner did not weigh himself last week”, but were otherwise de-identified. Reports included feedback about the patient’s most recent issues as reported during their IVR call, including shortness of breath, medication adherence, salt, and fluid intake, and increases in weight. CarePartners were asked to call their patient-partner weekly to review the reports and address identified problems.

CarePartners received guidelines about how to communicate in a positive motivating way, avoid conflict by respecting boundaries, include in-home caregivers, and respect confidentiality. Patients received a notebook including reminders and tips for their weekly patient-CarePartner calls. CarePartners received logbooks for tracking IVR reports, upcoming patient contacts, clinical encounters, and medication refills.

### Measurement

#### Baseline, 6-Month, and 12-Month Surveys

Patients’ HF-specific quality of life, self-care, and patient-CarePartner communication were measured via quantitative telephone surveys. Baseline sociodemographic variables included patients’ age, race, marital status, employment status, educational attainment, and income. Patients’ baseline depressive symptoms were measured using the 10-item version of the CES-D [42]. CarePartners completed online surveys at each time point; the current analyses include baseline CarePartner characteristics relevant to the comparability of groups at the time of randomization, and qualitative feedback from CarePartners’ 12-month surveys.

The primary outcome was HF-specific quality of life at 12 months, as measured by the Minnesota Living with Heart Failure Questionnaire (MLHFQ) [43]. HF self-care behaviors were measured using the Revised Heart Failure Self-Care Behavior Scale (HFSCB) [3]. A measure of HF medication adherence was created using the HFSCB adherence items with which patients reported how often they “took [their] pills every day”, “took [their] pills as the doctor prescribed, ie, took all of the doses of [their] pills”, “always refilled prescriptions for [their] pills on time”, and “had a system to help tell [them] when to take [their] pills”. The adherence measure based on these items was designed to identify patients reporting perfect adherence (ie, a binary measure identifying patients reporting “always” engaging in all four behaviors). Binary indicators for perfect adherence tend to correct for inflated adherence reporting [44,45].

To identify changes in patient-CarePartner communication, three relationship dimensions were measured at each time point. First, as an objective measure of communication intensity, patients were asked how often over the prior 6 months they communicated with their CarePartner by phone. Analyses examined patients’ likelihood of reporting that they spoke with their CarePartner at least twice per week. Second, the affective dimension of CarePartner support was measured using items based on prior studies of caregiving relationships [46,47]. Patients were asked how often they experienced each of six negative emotions when talking with their CarePartner (sadness, loneliness, anger, tension, guilt, or frustration), and analyses examined patients’ likelihood of reporting that they regularly experience one or more of these emotions. Third*,* to understand patients’ perspective of the difficulty involved in CarePartner communication, analyses examined participants’ likelihood of agreeing or strongly agreeing that it was “difficult to talk with [their] CarePartner about [their] illness”.

#### Weekly Interactive Voice Response Adherence and Symptom Reports

Patients’ IVR medication adherence and symptom reports were examined as potential indicators of differences across arms in intervention effectiveness, because short-term reporting intervals often provide information that is more reliable and less prone to bias than retrospective recall surveys [48-50]. Patients were considered adherent if they reported “always” taking their HF medication exactly as prescribed in the past week. Patients were classified as experiencing shortness of breath if they reported being bothered by shortness of breath “daily” or “several days” in the prior week. Patients were coded as having a significant weight gain if their reported weight met criteria described above. Finally, patients were coded as having positive self-reported health if they reported that their overall health was “excellent” or “very good”.

#### CarePartner Feedback

Although replies were not solicited to email reports sent to CarePartners based on the patient’s IVR feedback, if CarePartners did reply, that message was sent to the study coordinator. Also, in 12-month follow-up interviews, CarePartners were asked an open-ended question regarding what they felt were the strengths of the program. Here we briefly summarize both types of CarePartner feedback and include exhaustive lists of CarePartner comments in Multimedia Appendices 2 and 3.

### Statistical Analysis

The sample included all patients with 12-month surveys plus 22 patients for whom 6-month survey data were carried forward. Initial analyses compared the baseline characteristics of patients who did versus did not have 12-month data in the imputed sample. Subsequent analyses compared patients and CarePartners across arms in the sample with outcome data. IVR call completion rates were calculated using one record per week of attempted IVR calls, that is, 52 call-weeks per patient minus weeks in which the patient was on vacation or hospitalized. Logistic models were used to predict patients’ likelihood of completing each weekly call as a function of arm, baseline characteristics, and the number of weeks since enrollment. Statistical tests for the analyses of call completion rates were adjusted for clustering of call-weeks within patients.

The primary outcome was change in HF-specific quality of life between baseline and 12 months. The study was powered to detect a medium/small effect (*d*=.351) assuming a 20% loss to follow-up, similar to that observed in the prior HF trial by Sisk et al [51]. All outcomes were analyzed on an intent-to-treat basis. The xtmixed and logistic regression commands in Stata version 13.1 [52] were used to identify intervention effects on patients’ HF-related quality of life, self-care, and patient-CarePartner communication. Predictors for each analysis included an indicator for arm, time (baseline, 6-month, and 12-month), and an arm-by-time interaction. Effect estimates represent differences across arms adjusted for baseline values. To examine differences across arms in IVR reports of medication adherence and symptoms, graphical displays were created illustrating the proportion of patients reporting a given outcome each week, separately by arm. Logistic regression models were fitted to predict patients’ weekly IVR-reported outcomes, with weekly reports clustered within patient. Models included the following predictors: arm, time, and an arm-by-time interaction term. Variances for the estimated intervention effects were adjusted for the within-patient correlation of IVR reports across weeks [53-55]. To illustrate the magnitude of intervention-control differences in IVR-reported outcomes, the probability for each outcome at week 26 and 52 was predicted based on the logistic model separately for mHealth+CP and standard mHealth groups.

Among patients with chronic medical problems, depressive symptoms may influence their perceived health status even more than objective symptoms and impairments resulting from their medical condition [56]. Depressed patients often attribute their difficulties to insufficient social support [57,58], and CarePartners’ support may have counteracted their tendency to over-generalize health problems [59-61]. To test this hypothesis, we examined a potential interaction between patients’ baseline level of depressive symptoms (CES-D) and arm, with respect to IVR reports of excellent/very good health. Specifically, patients’ unadjusted frequency of reporting excellent/very good health was examined graphically as described above, within subgroups defined by baseline CES-D scores. Because graphical displays suggested an inflection point with two very different slopes, we fit logistic models separately for patients with CES-D scores that were low (0-4) versus high (5-10). Each model included terms for arm, baseline CES-D score, an arm-by-CES-D interaction, time, and an arm-by-time interaction.

### Human Subjects Approval

The study protocol was approved by the Ann Arbor VA Human Subjects Committee, and all patients provided written informed consent. Patients and CarePartners received US $20 for completed surveys; patients did not have financial incentives for completing IVR calls. None of the authors had any financial conflict of interest.

See Multimedia Appendix 4 for the CONSORT-EHEALTH checklist [62].

## Results

### Recruitment and Baseline Characteristics

**Figure 3:**
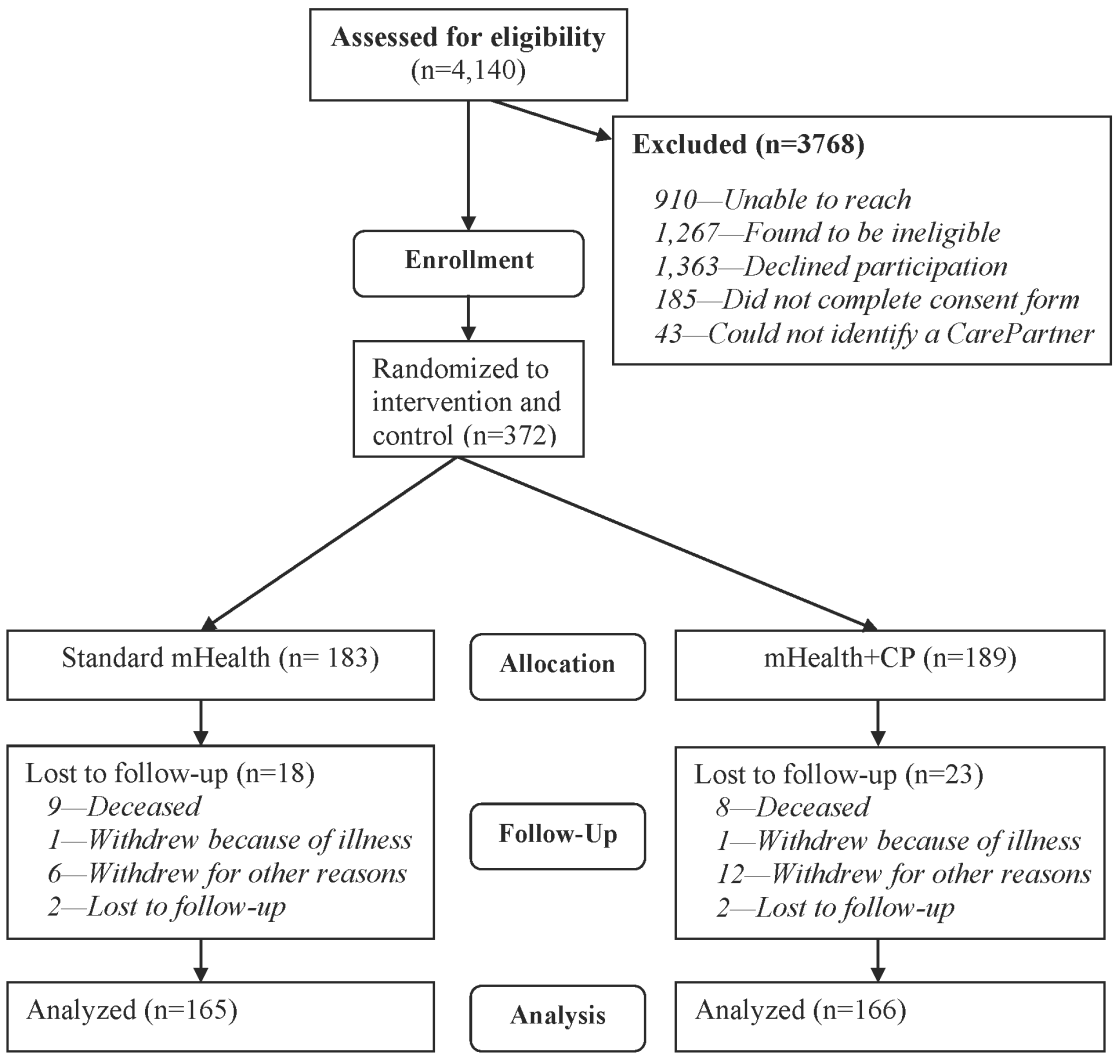
CONSORT Diagram for participants in the trial.

A total of 4140 potentially eligible patients were identified from electronic medical records. Of these, 372 were randomized, and 331 (89%) had outcome data at 12 months (see Figure 3). Patients lost to follow-up were less likely to report at baseline that they spoke with their CarePartner at least twice per week (43.9% versus 65.9%, *P*=.006) and had better baseline HF self-care scores as measured by the HFSCB (*P*=.002) but were not significantly different from patients with follow-up data on any other characteristic shown in Table 1 (see also Multimedia Appendix 5).

Patients in both arms had similar baseline characteristics, except that mHealth+CP patients were more likely to have a high school education or less (Table 1). Education was included in outcome analyses as a control variable, although analyses not including education as a covariate produced essentially the same results. There were no significant baseline differences across arms in measures of patient-CarePartner communication or in CarePartner characteristics. As expected in a VA population, most participants were male. Patients were on average 67.8 years of age (SD 10.2), 77.0% (255/331) were white, 48.0% (159/331) had a high school education or less, 32.6% (108/331) lived alone, and 87.6% (290/331) were retired or unemployed. While most patients (65.9%, 218/331) reported at baseline that they talked with their CarePartner by phone at least twice per week over the prior 6 months, 44.8% (147/328) reported regularly experiencing one or more negative emotions during those conversations, and 21.5% (71/331) agreed that it was difficult to talk with their CarePartner about their illness. Compared to patients, CarePartners were younger, more likely to be female, more likely to be employed, and had more years of education. A total of 41.4% (137/331) of CarePartners were the patients’ daughters/daughter-in-laws, 20.2% (67/331) were sons/son-in-laws, 11.2% (37/331) were friends, 9.1% (30/331) were sisters/sisters-in-laws, and the remaining 18.1% (60/331) were other family and social network members.

**Table 1:** Baseline characteristics of the sample.

		Overall (n=331)	Standard mHealth (n=165)	mHealth+CP (n=166)
**Patient characteristics**				
	Age in years, mean (SD)	67.8 (10.2)	68.1 (10.1)	67.6 (10.3)
	Male, % (n)	99.4 (329)	98.8 (163)	100.0 (166)
	White race, % (n)	77.0 (255)	77.0 (127)	77.1 (128)
	Married/Partnered, % (n)	58.9 (195)	61.2 (101)	56.6 (94)
	High school or less, % (n)	48.0 (159)	41.8 (69)	54.2 (90)
	Live alone, % (n)	32.6 (108)	32.7 (54)	32.5 (54)
	Unemployed/retired, % (n)	87.6 (290)	86.1 (142)	89.2 (148)
	Income <$15,000, % (n)	31.4 (104)	30.3 (50)	32.5 (54)
	CES-D Depression, mean (SD)	3.0 (2.5)	3.0 (2.5)	3.0 (2.5)
	MLHFQ^a^, mean (SD)	43.3 (25.3)	43.0 (26.4)	48.8 (24.3)
	HFSCB^b^, mean (SD)	82.8 (17.9)	82.6 (19.2)	83.0 (16.5)
	Adherent to HF Rx^c^,% (n)	52.3 (173)	50.3 (83)	54.2 (90)
**Relationship quality** ^d^ **, % (n)**				
	Talk 2+ times/ week	65.9 (218)	66.1 (109)	65.7 (109)
	Negative emotions^e^	44.8 (147)	45.4 (74)	44.2 (73)
	Perceived difficulty^f^	21.5 (71)	18.8 (31)	24.1 (40)
**CarePartner characteristics**				
	Age in years, mean (SD)	46.7 (13.2)	47.2 (14.5)	46.2 (11.9)
	Male, % (n)	35.0 (116)	32.7 (54)	37.3 (62)
	Married/Partnered, % (n)	68.6 (227)	67.3 (111)	69.9 (116)
	High school or less, % (n)	27.8 (92)	23.6 (39)	31.9 (53)
	Unemployed/retired, % (n)	36.9 (122)	38.8 (64)	34.9 (58)

^a^Minnesota Living with Heart Failure Questionnaire Scores. Lower scores indicate better functioning.

^b^Revised Heart Failure Self-Care Behavior Scale. Higher scores indicate better HF self-care.

^c^Percent of patients with perfect HF medication adherence over the prior month as measured by the four HFSCB items focused on adherence (see Methods).

^d^Patients’ reports regarding their relationship with their CarePartner.

^e^Percent of patients who report regularly experiencing any of six negative emotions when talking with their CarePartner (sadness, loneliness, anger, tension, guilt, or frustration).

^f^Percent of patients who agree that it is “difficult to talk to [their] CarePartner about [their] illness”.

### Interactive Voice Response Call Completion

Patients participated for a total of 15,709 call-weeks, during which they completed 14,175 calls, for a completion rate of 90.2%. IVR completion rates were essentially the same between mHealth+CP and standard mHealth arms (90.8% versus 89.7%), and there was no change in patients’ likelihood of completing IVR calls throughout follow-up (*P=*.19). The likelihood of call completion was unrelated to patients’ baseline HF-specific quality of life (MLHFQ) scores, HF self-management scores, CES-D scores, or measures of patient-CarePartner relationship quality (all *P* values ≥.15). IVR calls generated fax notifications to clinicians 1606 times (11.3% of completed calls), including 743 for weight gain, 774 for shortness of breath, and 89 for both problems. There were no differences in the number of fax alerts to clinicians between arms (*P*=.52).

### Intervention Effects

#### Effects on Quality of Life, Self-Care, and CarePartner Communication Measured via Surveys at 6 and 12 Months

There were no differences by arm at either 6 or 12 months in HF quality of life (MLHFQ) scores (Table 2; both *P*>.21). Overall, there were no differences by arm in HF self-care behaviors measured by the HFSCB composite score. However, based on the four HFSCB items addressing HF medication adherence, mHealth+CP patients were 8.8% more likely than standard mHealth patients to report taking medication exactly as prescribed at 6 months (62.8% versus 54.0%, *P=*.02) and 13.8% more likely at 12 months (66.4% versus 52.6%, *P*=.01).

**Table 2:** Intervention effects measured via 6- and 12-month surveys.

		Baseline to 6 months		Baseline to 12 months	
		mHealth+CP effect (95% CI)	*P* value	mHealth+CP effect (95% CI)	*P* value
**Quality of life and self-care**					
	MLHFQ^a^	+2.66 (-1.51 to 6.82)	.21	0.74 (-4.62 to 4.77)	.98
	HFSCB^b^	-2.33 (-6.00 to 1.35)	.21	-1.08 (-4.74 to 2.58)	.56
	Adherent to HF Rx^c^	+8.8% (1.2-16.5)	.02	+13.8% (3.7-23.8)	.01
**Relationship quality** ^d^					
	Talk 2+ times/ week	+10.2% (0.0-20.5)	.048	0.02% (-8.8%, 12.1%)	.76
	Negative emotions^e^	-9.9% (-19.8 to -0.1)	.049	-13.8% (-23.4 to -4.2)	.01
	Perceived difficulty^f^	-2.3% (-10.1 to 5.5)	.56	-8.3% (-16.6 to 0.0)	.049

^a^Minnesota Living with Heart Failure Questionnaire Scores. Lower scores indicate better functioning.

^b^Revised Heart Failure Self-Care Behavior Scale. Higher scores indicate better HF self-care.

^c^Patients’ likelihood of reporting perfect HF medication adherence over the prior 30 days as measured by the four HFSCB items focused on heart failure medication use (see Methods).

^d^Patients’ reports regarding their relationship with their CarePartner.

^e^Patients’ likelihood of reporting regularly experiencing any of six negative emotions when talking with their CarePartner (sadness, loneliness, anger, tension, guilt, or frustration).

^f^Patients’ likelihood of agreeing that it is “difficult to talk to [their] CarePartner about [their] illness”.

Patients’ survey responses indicated that dyadic communication with their CarePartner was more active and positive in the mHealth+CP arm. For example, in the 6-month survey, mHealth+CP patients had an absolute 10.2% greater likelihood than standard mHealth patients of reporting talking with their CarePartner at least twice per week over the prior 6 months (70.2% versus 60.0%; *P=*.048). mHealth+CP patients were significantly less likely than standard mHealth patients to report regularly experiencing negative emotions when talking with their CarePartner at the 6-month (31.9% versus 41.8%, *P=*.049) and 12-month follow-up (26.6% versus 40.4%, *P=*.01). Also, at the 12-month follow-up, mHealth+CP patients were 8.3% less likely than standard mHealth patients to agree that it was difficult for them to talk with their CarePartner about their illness (16.2% versus 24.5%; *P=*.049).

#### Effects on Adherence and Symptoms Reported Weekly via Interactive Voice Response

**Figure 4:**
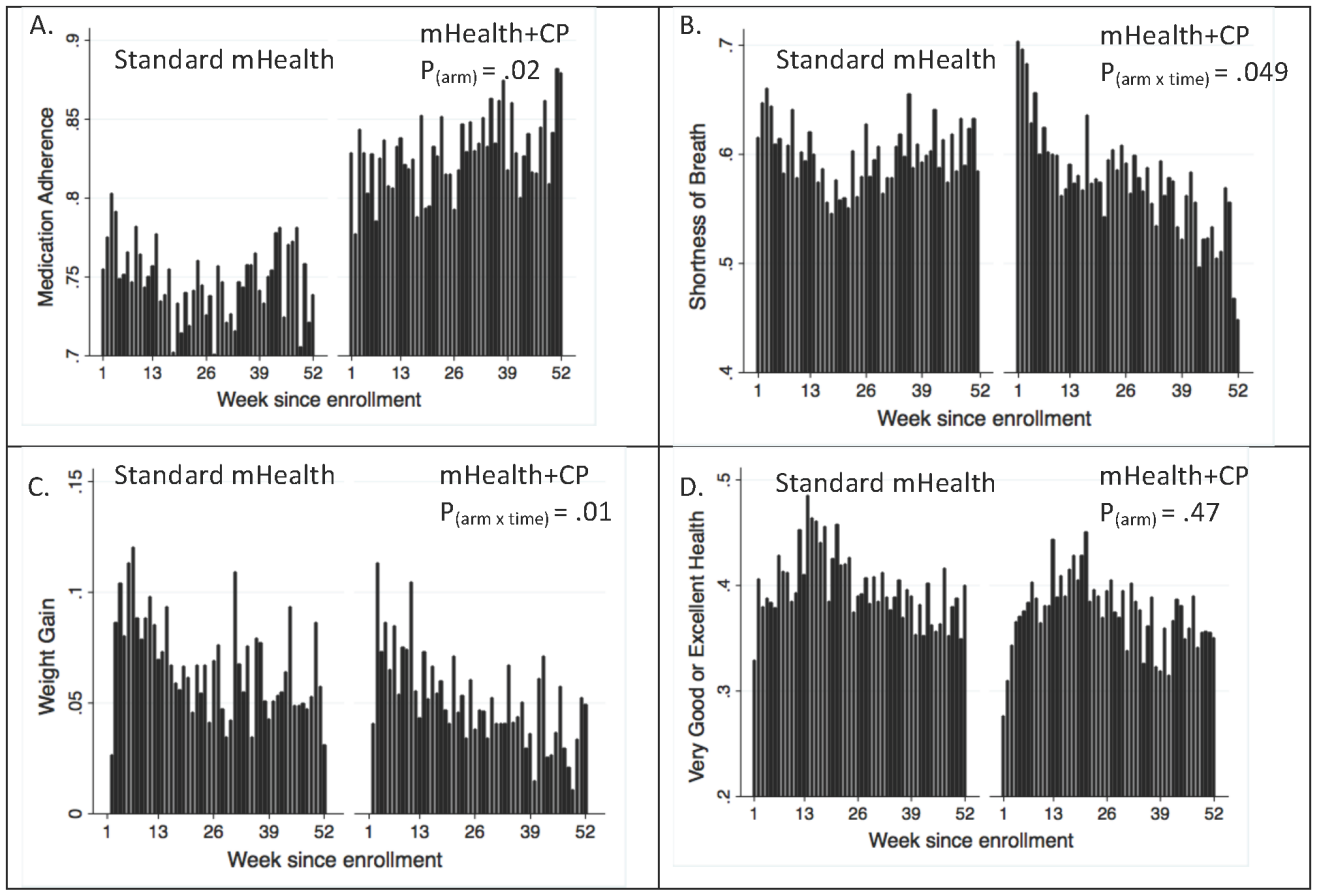
Unadjusted self-care and health status reports for patients in each randomization group by week since enrollment: Standard mHealth=patients randomized to IVR monitoring and self-care support with clinician alerts; mHealth+CP=patients randomized to the same intervention + weekly feedback to patients’ CarePartners. The Y-axis for each panel differs in scale; bars represent the proportion of patients responding with that report. P values are from logistic regression models testing differences across arms. P values <.05 represent significant effects favoring mHealth+CP. A: Reports of always taking heart failure medication exactly as prescribed in the prior week. B: Reports of being bothered by shortness of breath every day or several days in the prior week. C: Clinically significant weight gain generating a notification to patients’ healthcare team. D: Reports of very good or excellent health (versus good, fair, or poor health) in the prior week.

Displays of the unadjusted proportion of patients reporting perfect medication adherence, shortness of breath, and concerning weight changes via IVR suggested differences that favored mHealth+CP (Figure 4). These findings were substantiated by logistic regression analyses. Throughout the 1-year intervention, mHealth+CP patients were consistently more likely than standard mHealth patients to report perfect HF medication adherence over the prior week (main effect for arm, ie, ß=.5092; 95% CI 0.0857-0.9329; *P=*.02). There were no differences in time-trends in adherence reports across arms (*P=*.41), and the arm-by-time interaction term was excluded from the final model. Based on the logistic model, mHealth+CP patients had an 8.3% absolute greater likelihood of reporting perfect HF medication adherence in the prior week at 6 months (83.7% versus 75.4% for standard mHealth) and a 10.0% greater likelihood at 12 months (84.9% versus 74.9%).

Over the course of follow-up, mHealth+CP patients became increasingly less likely than standard mHealth patients to report shortness of breath during the prior week (arm-by-time interaction ß=-.0114; 95% CI -0.0206 to -0.0022; *P=*.049). The main effect of arm was not statistically significant (ß=.0894; 95% CI -0.2857 to 0.4644; *P*=.64). mHealth+CP patients had a 4% absolute reduction compared to standard mHealth patients in the likelihood of reporting shortness of breath at 6 months (57% versus 61%) and an 11.1% reduction at 12 months (50.1% versus 61.2%).

A significant arm-by-time interaction indicated that mHealth+CP patients were significantly less likely than standard mHealth patients to experience clinically significant weight increases (ß=-.0148; 95% CI -0.0232 to -0.0064; *P=*.01). The main effect of arm was not statistically significant (ß=.0454; 95% CI -0.2147 to 0.3055; *P*=.73). At 12 months, mHealth+CP patients had an absolute 2.4% decrease in the likelihood of generating a clinician notification for weight gain, relative to standard mHealth patients. Given the expected 12-month rate of significant weight increase in the standard mHealth group (5.4%), the reduction in the mHealth+CP arm represents a 44.4% relative improvement.

With respect to patients’ reports of excellent/very good health, arm had neither a main effect (ß=-.1469; 95% CI -0.5366 to 0.2427; *P*=.39) nor an interaction with time (*P*=.70).

#### Auxiliary Analysis of the Interaction Between Randomization Arm and Baseline Depression Scores With Respect to Perceived Health Status Reported via Interactive Voice Response

**Figure 5:**
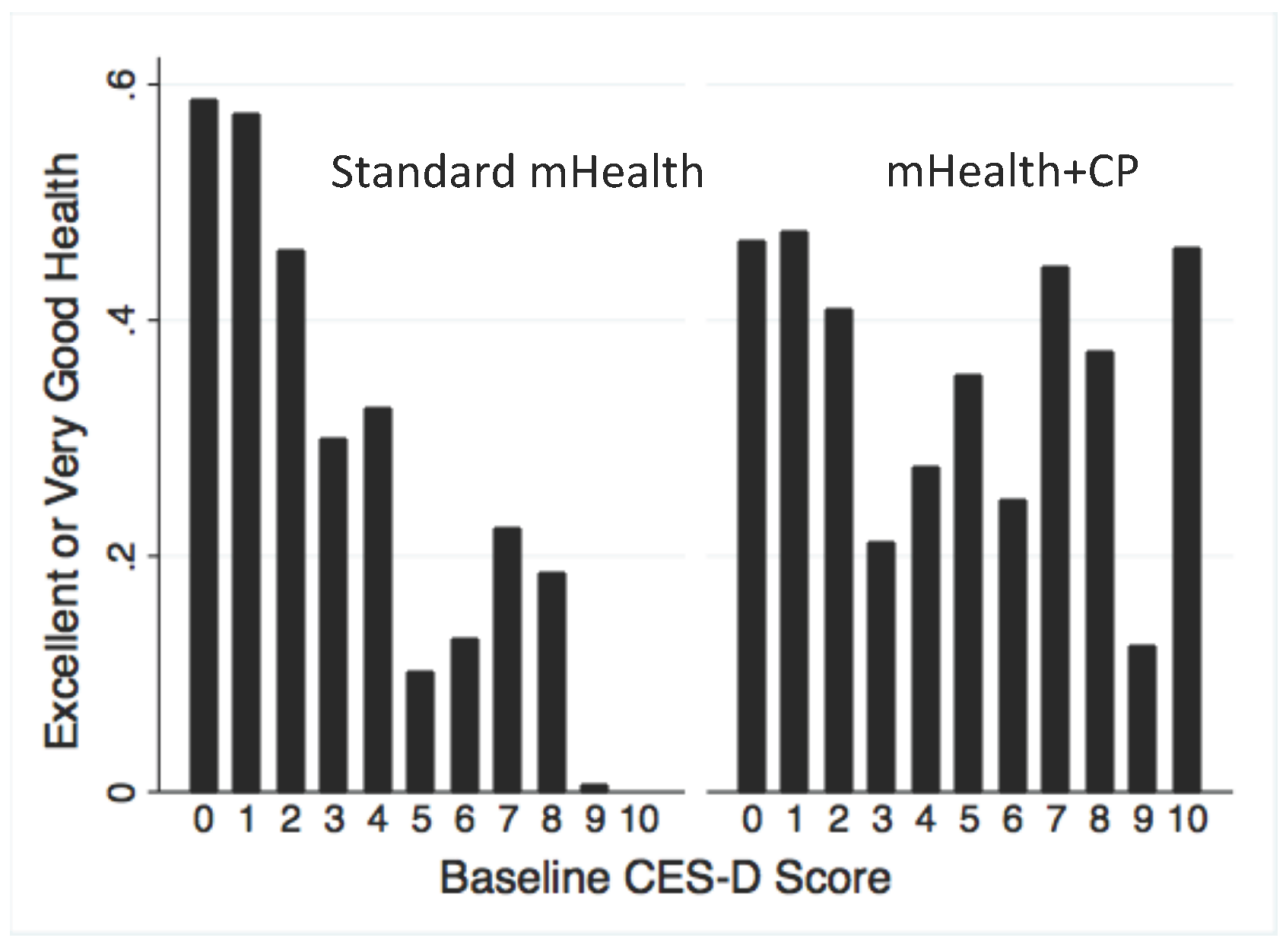
Unadjusted reports of excellent/very good health for patients in each randomization group by baseline CES-D depression score. Higher scores indicated greater depressive symptoms.

Among patients randomized to standard mHealth, there was a strong negative association between higher (ie, worse) baseline CES-D depression scores and patients’ likelihood of reporting excellent health via IVR (see Figure 5). In contrast, IVR reports of excellent health status were roughly constant in the mHealth+CP arm, regardless of the patient’s baseline level of depressive symptoms. The leveling of mHealth+CP patients’ perceived health reports across baseline CESD-levels reflected a somewhat lower proportion of mHealth+CP patients reporting excellent/very good health relative to standard mHealth patients when baseline CES-D scores were low, as well as a substantially higher proportion reporting excellent/very good health among those with greater baseline depressive symptoms. In multivariate analyses examining the effect of arm on patients’ likelihood of reporting excellent health status separately in groups with low CES-D (scores 0-4) and high baseline CES-D (5+) scores, the effect of mHealth+CP was significant in both groups. mHealth+CP had a positive effect among patients with higher baseline CES-D scores (ß=1.27; 95% CI 0.42-2.12; *P*<.01), and a smaller negative effect among patients with lower baseline CES-D scores (ß=-.46; CI -0.90 to -0.028; *P*=.04). According to these models, patients with a baseline CES-D score of 1 were 11% less likely to report excellent/very good health if randomized to mHealth+CP, while patients with a baseline CES-D score of 8 were 22% more likely to report excellent/very good health if randomized to mHealth+CP relative to the control group.

#### CarePartner Feedback

Although mHealth+CP CarePartners’ responses to IVR reports were not systematically tracked, many CarePartners did reply to those email reports, and their messages suggested that the structured alerts were read and acted upon (see Multimedia Appendix 2). Examples of text from those CarePartner replies include:

Hi. Thanks, there is nothing to report. He is doing quite well, thank you for your continuing caring and support.

Hi. [Patient-partner’s name] is coming alone fine, he was hospitalized for a few days due to an infection from his dialysis treatment, he is doing better today, he just returned from dialysis treatment. Thank you. 

Yes he has had a little shortness of breath and has sought council [sic] from his doctor. Thank you.

Qualitative feedback from mHealth+CP CarePartners in their 12-month online follow-up survey also suggested that they felt that the feedback about their patient-partner was useful and that they were using that information as the basis for a stronger, more active relationship related to their partner’s HF (see Table 3 for example quotes and Multimedia Appendix 3 for an exhaustive list of CarePartner comments). Comments suggest that CarePartners found the intervention useful not only for increasing the information base of their self-care assistance, but that it also served as a vehicle for strengthening their relationship with their patient-partner more generally.

**Table 3:** Example of responses to open-ended questions to mHealth+CP CarePartners in their 12-month follow-up survey regarding the perceived strengths of the program.

Category	Responses
Informational support and general knowledge about heart failure	I learned a lot about heart failure by being in the program. My father learned a lot too!
	[The program] gave me better insight into my dad’s health.
	It kept my relative in a reporting mode where he had to think about what he needed to do because someone would be checking in with him.
	I appreciated the weekly update regarding medications.
	[I liked] the CarePartner calls. The monitoring program is awesome.
	[I liked that] even if I hadn’t spoke with him yet, I knew from the email, he was ok.
Improved communication, reassurance, and relationship quality	[The program] helped my brother and I to get closer and communicate better.
	Communication about heart failure was more open.
	[The program] helped me understand my dad better.
	I liked that my dad told me a lot more about his health.
	I felt more comfortable talking to my brother about his heart failure.
	[The program] helps me to keep in touch with my cousin on a regular basis.
Ease of use and general positive comments	[mHealth+CP was] friendly, easy to understand, the questionnaire was easy to navigate.
	It was not very intrusive.
	As far as what I liked about the program, the fact that it even exists! It a wonderful idea and hopefully will yield results that are helpful to your patients.
	I think it made my Dad a little more responsible because he was more accountable to an outside party.

## Discussion

### Principal Findings

In this randomized comparative effectiveness trial, no group differences were identified at 6 or 12 months in the primary outcome of HF-specific quality of life or the composite measure of HF self-care. However, a number of potentially important differences in the process and outcomes of care were identified that favored mHealth+CP compared to standard mHealth. For example, in both follow-up surveys, a greater proportion of mHealth+CP patients reported perfect medication adherence, and mHealth+CP patients were consistently more likely throughout the 1-year follow-up to report via IVR that they took their HF medications as prescribed during the prior week. mHealth+CP patients also had a significantly greater decrease in their likelihood of reporting shortness of breath via IVR and were less likely to report clinically significant weight gains.

The strongly negative association between patients’ baseline depressive symptoms and IVR reports of perceived general health that we observed in the standard mHealth group was not apparent among patients who were randomized to mHealth+CP. In particular, patients with more severe depressive symptoms at baseline were relatively likely to make positive self-assessments about their health via IVR if they were in the mHealth+CP arm. This finding (as well as the feedback from CarePartners presented here) is consistent with studies suggesting that social support can have powerful impacts on patients’ well-being over and above the concrete benefits in terms of specific self-management behaviors [58].

These intervention effects represent positive impacts in some of the most fundamental areas of HF-related self-care and morbidity. Medication adherence is vital for HF patients, and poor adherence is a major predictor of acute events [63]. Shortness of breath and rapid weight gain are correlates of patients’ functional decline and used as sentinel events to identify patients at high risk for acute episodes. If these risk factors can be effectively addressed via mHealth services such as this one that focus on increasing caregiver support instead of the use of costly medical services, it would represent a major advance.

It is important to emphasize that these intervention effects were observed in a comparative effectiveness trial, over and above potential changes in health and self-care among patients receiving an active control intervention. All participants identified a CarePartner prior to randomization, and control patients and CarePartners received considerable information about HF self-care and self-management support. Control patients also received weekly IVR monitoring and self-management support calls with feedback to their clinician.

A soon-to-be-published companion paper using survey data from CarePartners in this same trial provides additional positive information consistent with the patient information reported here [38]. Compared to CarePartners in the standard mHealth arm, those randomized to mHealth+CP reported greater involvement in the patient’s medication adherence at both endpoints (both *P*<.05). mHealth+CP CarePartners also were more likely to report attending the patient’s medical visits at the 6-month follow-up. Importantly, CarePartners reporting the most symptoms of depression and strain at baseline had those symptoms significantly reduced if the CarePartner was in the mHealth+CP versus standard mHealth arm. These CarePartner reports as well as the qualitative feedback from CarePartners reported here suggest that involvement in mHealth information exchange may significantly improve relationship quality and self-management assistance for patients with chronic health problems. The qualitative feedback presented in Table 3 and Multimedia Appendix 3 is particularly interesting—since many mHealth+CP CarePartners volunteered that the intervention served to strengthen their relationship with their patient-partner.

The 1-week reporting interval used for the IVR-based outcome measures may have been more sensitive to intervention effects than the 6- and 12-month surveys. Differences across arms in IVR-reported adherence were consistent over the 12-month follow-up, and improvements in shortness of breath and weight became evident only after several months of program participation. This suggests that the pattern of effects is not the result of biased reporting, which tends to be immediate and short-lived [64]. Also, improvements in health and self-care measured via IVR were consistent with patients’ improved medication adherence reported in both follow-up surveys and with reports of more frequent supportive communication with CarePartners. More generally, reports of health behaviors are more reliable when reporting intervals are brief, avoiding the biases associated with longer periods of retrospective recall [44,45,49,50,65].

### Limitations

This trial had several limitations. It is possible that patients were biased about their medication adherence reporting in order to avoid burden for their CarePartner or conflict in the relationship. However, prior studies have shown that patients’ medication self-reports are highly correlated with objective measures of medication use, especially when the recall interval is short and the measure is designed to identify even mild forms of non-adherence [44,45]. Also, other positive reports from both patients and CarePartners in this trial corroborate patients’ IVR reports of medication adherence when randomized to mHealth+CP. Nevertheless, it would be important to confirm these findings with medication refill data. Similarly, it would be useful to verify patients’ self-reported weights using data-storing electronic scales. Another limitation is that the trial was conducted among VA patients, nearly all of whom were men. Caregiving dynamics differ by patients’ demographic and clinical characteristics, and future studies should determine whether results can be replicated in other populations, including non-VA patients and women. Some important clinical information about participants was not collected during the trial. For example, we do not know whether patients underwent cardiac surgery, resynchronization therapy, or revascularization. While we have no indication that randomization was unsuccessful, and patients in both groups were well matched on a wide range of baseline characteristics, it remains possible that unobserved differences in patients’ clinical status at the time of enrollment may have contributed to the intervention effects observed. Finally, our study had several outcomes measured at two time points, and multiple comparisons may have contributed to the findings. However, results were consistent with the study’s theoretical framework, and significant results were consistently in the same direction, that is, favoring mHealth+CP over standard mHealth.

### Conclusions

This comparative effectiveness trial suggests that, although not all outcomes were different across arms at follow-up (notably HF-specific quality of life and a composite measure of HF self-care), providing caregivers with automated updates and guidance on self-care support may enhance the beneficial effects of mHealth for HF patients’ health and self-management. Given increasing numbers of patients with chronic illness and the growing strains on clinical resources, health systems using mHealth approaches should consider creative ways to engage patients’ social supporters to play a more active role. Finally, trials such as this one that include frequent mHealth monitoring may uncover intervention effects that are missed through more intermittent surveys and lengthy retrospective recall intervals.

## Appendix

Multimedia Appendix 1 Operationalization of inclusion and exclusion criteria.

Multimedia Appendix 2 Unsolicited email replies from CarePartners when receiving feedback about the status of their patient-partner.

Multimedia Appendix 3 Responses to open-ended questions to mHealth+CP CarePartners in their 12-month online follow-up survey regarding the perceived strengths of the program.

Multimedia Appendix 4 CONSORT-EHEALTH checklist V1.6.2 [65].

Multimedia Appendix 5 Comparison of baseline characteristics for patients with and without follow-up data.

## Abbreviations

HF: chronic heart failure
HFSCB: Revised Heart Failure Self-Care Behavior Scale
IVR: interactive voice response
HSR&D: Health Services Research & Development
mHealth: mobile health
mHealth+CP: mobile health plus CarePartner
MLHFQ: Minnesota Living with Heart Failure Questionnaire
NSSQ: Norbeck Social Support Questionnaire
VA: Veterans Health Administration
