# Supplementary material for: A Mobile Health Intervention Supporting Heart Failure Patients and Their Informal Caregivers: A Randomized Comparative Effectiveness Trial
Source: J Med Internet Res. 2015 Jun 10;17(6):e142. doi: 10.2196/jmir.4550 (PMC4526929; doi:10.2196/jmir.4550)
Supplement: Multimedia Appendix 1 [file jmir_v17i6e142_app1.pdf]

Multimedia Appendix 1. Operationalization of Inclusion and Exclusion Criteria

Electronic Inclusion criteria:

Include patients with:

a. Diagnosis of heart failure (New York Heart Association classification II-III) and
b. Patients who have had an out patient visit within the last 12 months

ICD-9 CODES FOR HEART FAILURE: CHF PATIENT RECRUITMENT

428.0
428.1
428.9
428.3
428

402.0-402.9 (w/a fifth-digit 1), or 404.0-404.9 (w/a fifth-digit 1 or 3), and 428.0-428.9.

Electronic Exclusion Criteria:

Exclude patients with any of the following:

1. End stage renal disease (codes 4031 or 4042 or 4043) OR
2. Lung cancer (codes 1990) OR
3. Dementia (codes 0461, 0463, 2900, 29010, 29011, 29012, 29013, 29020, 29021, 2903, 29040, 29041, 29042, 29043, 2908, 2909, 3310, 3311, 3312, 3317, 3319) OR
4. Bipolar (codes 2960, 2961, 2964, 2965, 2966, 2967, 2968) OR
5. Schizophrenia (codes 2950, 2951, 2952, 2953, 2954, 2956, 2957, 2958, 2959)

CHF patients with most recent ejection fraction > 40%

Non-Electronic Exclusion Criteria:

Phone screening: exclude if any of the following:

- Can’t speak English
- Limited life expectancy (advanced stage cancer, heart failure, on oxygen, end stage renal disease).
- Not planning to get all or most of care at study site.
- PCP isn’t affiliated with study site.
- Alcohol problems
- Illegal drug use
- Receiving palliative care due to advanced HF or other health problems
- Unable to use a telephone to respond to weekly automated self-management support calls
- Unable to nominate an eligible CP

For the CarePartner Inclusion Criteria:

- Must live outside of the patients home, at any distance within the United States
- Must have access to a computer with an Internet connection, either at home or through some other location
- Have ability to communicate via e-mail
- CPs must report talking with the patient by phone or in-person at least once per month over the prior 6 months
- Must agree with additional criteria for patient involvement

For the CarePartner Exclusion Criteria:

- Have a serious mental illness (bipolar/schizophrenia)
- Cannot speak English
- Less than 21 years of age
- No working telephone in their home
