# Supplementary material for: A Mobile Health Intervention Supporting Heart Failure Patients and Their Informal Caregivers: A Randomized Comparative Effectiveness Trial
Source: J Med Internet Res. 2015 Jun 10;17(6):e142. doi: 10.2196/jmir.4550 (PMC4526929; doi:10.2196/jmir.4550)
Supplement: Multimedia Appendix 2 [file jmir_v17i6e142_app2.pdf]

Multimedia Appendix 2. Unsolicited email replies from CarePartners when receiving email feedback about the status of their patient-partner.

Thank you, yes [my patient-partner] has been doing fine on his diet, missing the foods that he loves but has to stay away from them.

Hi, yes he has had a little shortness of breath and has sought council from his doctor. Thank you.

Hi there, sorry I missed your message. He is doing fine, his weight is 136 lbs, and he says he feels just fine. Thanks for keeping in touch with us.

Hi thanks there is nothing to report he is doing quiet well thank you for your continuing caring and support.

[After missed call]: He was out in the sunshine with his wife. Thank you.

My dad had a routine VA follow up appointment yesterday that conflicted with this phone call. I thought he was going to call yesterday morning to advise this. I heard his appointment went very well with no issues. Thanks.

Hi he has contacted his MD for further instructions. Thanks a lot.

My father is travelling and I expect he'll make the call this week. There isn't any health problem. Thanks.

My brother has trouble answering the phone and pushing the buttons. His wife is with him almost constantly. I am in regular contact, every few days. If there is ever a problem with him, his wife would let me know immediately.

[After 2 weeks of missed calls]: Ok, I'm on it.

He is in the hospital recovering from removal of a benign brain tumor on [date]. He is currently in the ICU and when released from the hospital he will be going to rehab.

Hi. He is doing just fine and going on with his busy day to day. I just talked to him he is out on the town with the senior form.

Hi. He is coming alone fine, he was hospitalized for a few days due to an infection from his dialysis treatment, he is doing better today he just returned from dialysis treatment. Thank you.

Hi. He is better now his infection is clearing and he's feeling better.

He is okay. He thinks he [missed his call because he] slept through the phone ringing.

He is doing fine, he was still at dialysis appt. Thank you.
