# Supplementary material for: A Mobile Health Intervention Supporting Heart Failure Patients and Their Informal Caregivers: A Randomized Comparative Effectiveness Trial
Source: J Med Internet Res. 2015 Jun 10;17(6):e142. doi: 10.2196/jmir.4550 (PMC4526929; doi:10.2196/jmir.4550)
Supplement: Multimedia Appendix 3 [file jmir_v17i6e142_app3.pdf]

Multimedia Appendix 3. Responses to open-ended questions to mHealth+CP CarePartners in their 12-month online follow-up survey regarding the perceived strengths of the program.

Informational Support and General Knowledge about Heart Failure.

I learned a lot about heart failure by being in the program. My father learned a lot too!

[The program] gave me better insight to my dad's health.

If I had any questions, they would be quickly answered.

[mHealth+CP email feedback] provided information about my father's illness.

I liked receiving literature on heart failure.

This helped my relative understand and pay closer attention to his CHF--how important it is to monitor his symptoms.

I liked the information.

[The program] helps you learn how to take care of your health.

[I liked] making sure my father was healthy, until I felt he was in better shape.

[I liked the] support and information.

[The program] provided resources if needed as to where to go for information.

Lots of information.

[I liked] the information received in regard to heart failure.

A lot of the questions made me do or think about my partner's health.

I learned what to look for and how to help my dad with HF.

I felt that there was always someone I could get information from if needed.

[The program] gave me things to look for.

The staff were always willing to help and answer questions.

The staff gave me ideas of ways to help my relative.

The program lets you follow up often on the health progress of your partner.

[The program] made me think.

[I like] the help and information I received.

[I liked the] initial information packets.

It was helpful in giving much needed information about heart failure.

It helped me to know what to look for with any changes in my partner's health.

Information.

[I liked] learning about heart failure and what to look for. The newsletters were very informational and easy access.

My partner gained a better understanding about his condition, and his outlook regarding his future has improved.

I liked the information packet.

It kept my relative in a reporting mode where he had to think about what he needed to do because someone would be checking in with him.

I liked the fact that you called weekly to check on his condition and then informed me.

Thought provoking.

[I liked] being aware of what to do.

I appreciated the weekly update regarding medications.

Communication and information.

[I liked the] educational information given on website.

CHF awareness and monitoring.

We were very informed regarding heart failure.

[The program] made me aware of my dad’s condition.

It was informational.

Information.

I liked the weekly check-ins.

[I liked the] personal information for better healthcare.

It was good to know that the VA hospital cared about their veterans. I love telling my brother things that I have learn about how to care for him. Thank you for all the insight.

[I liked] the CarePartner calls. The monitoring program is awesome.

Nice to understand what exactly it is and how you can help.

[The program] gave good start ideas on heart care.

[I liked] the newsletters.

[The program] helped me be informed and what to do to help.

It was a positive approach to helping my relative.

[I liked] the updated information and the weekly phone calls.

The information about fluid intake [was useful].

[I liked] the updates via mail.

[I liked the] weekly report follow-up emails.

[The program] helped me deal with the health issues with more understanding.

The packet of information given at the beginning of the program [was useful].

[The program was] very informative on the disease.

[I liked] the weekly e-mail updates.

[The program] made me look for the smaller things like signs of depression.

The surveys allowed me to think on a lot of things that ordinarily I would not have, and how to deal with my dad more effectively.

I got a better understanding of heart failure.

[I liked] being more connected to the care of my partner, and being kept up to "speed" on how they're feeling and doing those times that I can't make it over to his/her house.

My relative is more aware of his problem.

Being helpful.

I liked that I was given ideas on what I should be paying attention to concerning my relative's health. It gave me a good reason to try and bring up my relative's health with him. I would say we need to discuss your health a little so I know how to answer the surveys, and sometimes he would actually talk to me about his health. My relative doesn't like to talk or complain about his health to anyone so it makes it difficult to discuss issues with him.

[I liked] getting to know about my friend's health problem better.

[I liked the] e-mails about him.

Information.

I liked the availability of information.

[I liked] the level of detail.

[The program] has made me realize specific issues my father faces.

Gives me a little insight as to what signs to look for with his medical problems.

The weekly updates were great.

[The program] taught me how heart failure can affect a person's life. How I could do things to help, and also, understand what they might need help doing.

They keep me up to date on medical issues.

I appreciate that they gave me feedback about my patient's feelings through a 3rd party.

[The program was] informative about the needs of my CarePartner.

The CarePartner program helped bring to the forefront the importance of monitoring diet, weight, medications, ADL's, around CHF.

[I liked] getting weekly updates.

It keeps me informed.

[I liked] knowing my dad's weight.

[I liked that] even if I hadn't spoke with him yet that week, I knew from the email, he was ok.

<u>Improved Communication, Reassurance, and Relationship Quality.</u>

I felt good about my visits.

[I liked] the support it has given me.

I liked that I was able to give my input.

[The program] helped my brother and I to get closer and communicate with each other better.

I felt as though my father was as important to this program and yourselves as he is to me and my family. I can appreciate all the time and effort that was put into my father.

[I liked] having someone to talk to about my family member's health.

[I liked] the contact of keeping up on what health situation my uncle is in.

The support was EXCELLENT!

[I like] that you keep close tabs on my brother by calling and checking on him regularly.

Good idea. I am a clinical dietitian who counsels patients with CHF, diabetes, cardiac patients, etc. The emotional support is so very important.

I enjoyed the fact that he asked me to be his CarePartner. He is a very private person who doesn't like to be told what to do.

Communication about heart failure was more open.

[I liked] getting more involved in my family member's life.

[I liked] finding out different information to talk about with my relative.

[I liked the] contact and advice.

I got a better understanding of my partner and what they were dealing with.

My relative is being taking care of.

Improved communications.

[The program] made me think about the other people in my life and what they need.

[The program] helped me understand my dad better.

[The program] encouraged me to talk and ask questions about his condition.

I learned more about his heart failure and interacted more with him.

[The program] brought us closer.

I liked talking with my CarePartner.

I ask more questions now.

[I like the] communication you have with me and my relative.

It reminded me of specific things to check with my partner about.

It provided a weekly update and opportunity for discussion.

I liked that my dad told me a lot more about his health.

It let me define to myself our real relationship.

I enjoyed becoming closer to my brother. He is now letting me help him. Thank you!

It kept me motivated to inquire about my friend's health.

It made me think more about talking about heart failure as a distinct topic rather than just talking about his health.

[The program] helped open up a relationship with myself and my father concerning his help.

[The program] makes me stay in touch with my friend's health.

[I liked the] accountability my father felt about his health care.

It opened up for more contact and closeness.

I appreciated the better understanding of my Dad's illness and a closer relationship as an added benefit.

It was very informative and I fell more comfortable talking to my brother about his heart failure.

[I liked] the contact with my dad.

It reminded me to make an effort to offer long distance support.

[I liked] working more closely with [my patient-partner].

I stayed in contact.

It forces me to be more aware and attentive to my partner's needs and circumstances.

[I liked] knowing I might help my brother some with his health.

I liked talking more with my dad. Usually it's my mom that I would talk with.

I liked the surveys because they helped to give me ideas on things I can ask him about and things we can work on together to help him be happier and healthier.

I like that you try and have your patients become more open and close with their relatives.

Talking.

[The program] encouraged communication.

[I liked] how close I have become to my parents.

[I liked] getting a little closer to dad and learning more about his illness.

It brought us closer together!!

[The program] gave ideas for conversation.

I got closer to my husband's health issues. He confided in me and his condition and made me feel better. I feel closer to him.

Accountability

We talked about his problems and also mine. We sort of helped each other get a grip on them and face the music.

[I liked the] chance to know what is going on.

[The program] helps me to keep in touch with my cousin on a regular basis.

[I appreciated] being involved with my dad's care.

[The program] reminded me to check in with my dad concerning his health.

[I appreciated] the accountability he has with this, me and with the program seem to encourage him to be open.

The reassurance to keep you in contact with your relative.

I appreciated becoming closer to my dad and feeling like he truly trusts me in everything.

[I liked the] opportunity to participate with my father.

I liked receiving the weekly emails to know what my partner reported to you. I like that it provided another opportunity to discuss his health with him.

[I liked] getting closer to my brother

[I liked that the program was] checking on my dad weekly and letting me know that they talked to him.

[I liked] discussion with [my patient-partner].

I like the weekly emails verifying the status call.

[The program] made me talk to my Dad more about his health.

I was connected more with [my patient-partner].

[I liked] the time you spent on the phone with my father and the questions you asked to make me rethink my position.

By taking your survey I now see things with my dad I did not see before. If something does not feel right to me, we take fast action.

I was able to keep up with my dad's progress.

It brought us closer.

You have been there for [my patient-partner].

[I liked] sharing it with Dad.

Great program. It kept my father active and alert to his weekly phone calls.

Keeping me informed of his weekly calls from the program. Letting me know his progress and/or problems.

A new way to look at this problem.

It seemed that the program is concerned about patient care and the family.

I truly think you all really care.

The concern.

Keeping in touch.

Communication.

Thinking about my brother weekly.

They are helping my father.

They seem to care.

Concern.

They care.

The pleasant friend I grew upon.

You are persistent in checking up on the care of [my patient-partner] and are concerned with his welfare.

It opened a door for my dad to feel like he needed to and/or wanted to share more health history with me.

It provided a reminder to contact the partner more often to let them know we care.

<u>Ease of Use and General Positive Comments.</u>

The program was very interesting.

The life of my friend/relative has been greatly improved.

[mHealth+CP was] friendly, easy to understand, questionnaire was easy to navigate.

They were very consistent.

The program was easy to use.

Everything was nicely presented.

It was very easy to participate.

The information was easy to read and understand.

I liked the online accessibility.

It was not very intrusive.

It was a help to my dad.

You stay in touch.

Everyone is very friendly.

I was able to denote a problem.

As far as what I liked about the program, the fact that it even exists! It a wonderful idea and hopefully will yield results that are helpful to your patients.

I found everything excellent.

[I liked] helping my friend.

The weekly follow up calls to my dad and the email alerts stating they made those calls to my Dad. How my Dad was engaged in the program.

The program keeps them on our mind... life can be hectic…even for seniors.. E-mail notifiers help.

[I appreciated] your concern for [my patient-partner].

[I like] how you are concerned with the patients' health, and I like how you check up on a person.

[The program] helped my mom, who was the primary caretaker.

[I appreciate] that there is an organization such as yours.

[The program] helped me really appreciate my Dad's wife. She is his major support.

The program made the patient expect and respond to a call once a week.

I think my dad looked forward to your questions.

I think it made my Dad a little more responsible because he was more accountable to an outside party.

[I liked] that they called weekly for updates.

[I liked] the phone calls to my partner and the e-mails I receive concerning his health.

[I liked] reminds me to ask more questions to him on health issues.
