# Supplementary material for: A Mobile Health Intervention Supporting Heart Failure Patients and Their Informal Caregivers: A Randomized Comparative Effectiveness Trial
Source: J Med Internet Res. 2015 Jun 10;17(6):e142. doi: 10.2196/jmir.4550 (PMC4526929; doi:10.2196/jmir.4550)
Supplement: Multimedia Appendix 4 [file jmir_v17i6e142_app4.pdf]

CONSORT-EHEALTH Checklist V1.6.2 Report

(based on CONSORT-EHEALTH V1.6), available at [http://tinyurl.com/consort-ehealth-v1-6].

4550

Date completed

4/8/2015 15:56:48

by

Piette

A Mobile Health Intervention Supporting Heart Failure Patients and Their Informal Caregivers: A Randomized Comparative Effectiveness Trial

TITLE

1a-i) Identify the mode of delivery in the title

"A Mobile Health" Intervention Supporting Heart Failure Patients and Their Informal Caregivers: A Randomized Comparative Effectiveness Trial

1a-ii) Non-web-based components or important co-interventions in title

A Mobile Health Intervention Supporting Heart Failure Patients "and Their Informal Caregivers": A Randomized Comparative Effectiveness Trial

1a-iii) Primary condition or target group in the title

A Mobile Health Intervention Supporting "Heart Failure Patients" and Their Informal Caregivers: A Randomized Comparative Effectiveness Trial

ABSTRACT

1b-i) Key features/functionalities/components of the intervention and comparator in the METHODS section of the ABSTRACT

"Methods. 331 HF patients identified a “CarePartner” outside their household and received weekly interactive voice response (IVR) calls, i.e., “standard mHealth.” CarePartners of patients randomized to “mHealth+CP” received emails based on patients’ IVR responses."

1b-ii) Level of human involvement in the METHODS section of the ABSTRACT

CarePartners of patients randomized to “mHealth+CP” received "automated emails" based on patients’ IVR responses.

1b-iii) Open vs. closed, web-based (self-assessment) vs. face-to-face assessments in the METHODS section of the ABSTRACT

Self-care and symptoms were measured via 6- and 12-month surveys "with a research associate", and were measured weekly "via IVR."

1b-iv) RESULTS section in abstract must contain use data

"331 HF patients" identified a “CarePartner” outside their household and received weekly interactive voice response (IVR) calls, i.e., “standard mHealth.” CarePartners of patients randomized to “mHealth+CP” "(N=166)" received automated emails based on patients’ IVR responses.

1b-v) CONCLUSIONS/DISCUSSION in abstract for negative trials

The trial was not negative.

INTRODUCTION

2a-i) Problem and the type of system/solution

Research has demonstrated the utility of informal caregivers in helping chronically-ill patients follow self-management recommendations by providing support that is unavailable through professional care management [17-20]. However, in-home caregivers are often elderly, ill, and overwhelmed [21, 22]. Most in-home caregivers lack the training and resources needed to systematically monitor HF patients and provide self-management assistance. Moreover, chronically-ill patients increasingly have caregivers outside of the household, making health and self-care monitoring much more difficult [23, 24].

The CarePartner program was developed to address these challenges by enabling structured support by informal caregivers (CarePartners) who reside outside the patient’s home. Through this program, patients receive regular monitoring and tailored self-management education via IVR calls with feedback to their clinician. Prior studies demonstrated that chronically-ill patients are able and willing to regularly complete IVR calls and report clinically meaningful information [25, 26]. While one large trail of HF patients found no impact of IVR telemonitoring on rehospitalization or death post-discharge [27], meta-analyses have shown that remote patient monitoring with clinician feedback and self-management assistance can improve the quality and outcomes of HF care [28, 29]. It remains unclear, however, whether feedback to CarePartners is helpful over-and-above the support provided directly to patients and clinicians.

2a-ii) Scientific background, rationale: What is known about the (type of) system

Telephone care management can improve HF patients’ prognosis [6-10]. However, telephone follow-up is inadequately reimbursed and competes with in-person care for clinicians’ time [11]. Mobile health (mHealth) interventions, including interactive voice response (IVR) calls, can improve self-care behaviors and physiologic risk factors [12-15]. But without systems to follow-up on identified problems, increased monitoring may be insufficient to fill the gap between what HF patients need and what health systems can provide [15, 16].

METHODS

3a) CONSORT: Description of trial design (such as parallel, factorial) including allocation ratio

The current study reports the results of a randomized comparative effectiveness trial testing the impact of systematic feedback to patients’ CarePartners, compared to patients receiving standard mHealth monitoring and self-management education. Analyses focused on changes in patients’ HF-related quality of life, self-care, and patient-CarePartner communication reported via 6- and 12-month surveys, as well as on patients’ medication adherence and symptoms reported via weekly IVR calls.

3b) CONSORT: Important changes to methods after trial commencement (such as eligibility criteria), with reasons

Not applicable.

3b-i) Bug fixes, Downtimes, Content Changes

Not applicable.

4a) CONSORT: Eligibility criteria for participants

Patients were recruited from VA Cleveland Medical Center outpatient clinics between June 2009 and January 2012 and were followed for 12 months. To be eligible, patients had to: have a HF diagnosis, New York Heart Association classification of II or III, and a documented ejection fraction <40% (see Electronic Supplementary Material 1 for details). Patients also had to have attended at least one VA outpatient visit within the previous 12 months, have a VA primary care provider, and be able to participate in automated telephone calls in English. Patients also needed to nominate an eligible CarePartner, i.e., a relative or friend living outside their home. Patients were excluded if they: lived in a skilled nursing facility; were prescribed oxygen supplementation; were receiving palliative care; had a life-threating condition such as lung cancer; or had ICD-9 coded diagnoses indicating dementia, bipolar disorder, or schizophrenia.

4a-i) Computer / Internet literacy

Computer literacy was not a criterion for eligibility.

4a-ii) Open vs. closed, web-based vs. face-to-face assessments:

Potentially eligible patients identified from electronic medical records were sent an invitation letter, followed by a screening and recruitment call. Eligible and interested patients were mailed informed consent forms and were assisted in identifying potential CarePartners using the Norbeck Social Support Questionnaire (NSSQ) [30].

4a-iii) Information giving during recruitment

The informed consent form will be submitted as an appendix.

4b) CONSORT: Settings and locations where the data were collected

Patients were recruited from VA Cleveland Medical Center outpatient clinics and were followed for 12 months.

Patients' HF-specific quality of life, self-care, and patient-CarePartner communication were measured via quantitative telephone surveys.

4b-i) Report if outcomes were (self-)assessed through online questionnaires

Weekly IVR Adherence and Symptom Reports. Patients' IVR medication adherence and symptom reports were examined as potential indicators of differences across arms in intervention effectiveness, because short-term reporting intervals often provide information that is more reliable and less prone to bias than retrospective recall surveys [39-41].

4b-ii) Report how institutional affiliations are displayed

Not applicable.

5) CONSORT: Describe the interventions for each group with sufficient details to allow replication, including how and when they were actually administered

5-i) Mention names, credential, affiliations of the developers, sponsors, and owners

We note that the authors investigators have no conflict of interest and there were no other system developers.

5-ii) Describe the history/development process

The CarePartner program was developed to address through a series of pilot and feasibility studies with VA and non-VA patients to address these challenges by enabling structured support by informal caregivers (CarePartners) who reside outside the patient's home.

5-iii) Revisions and updating

Not applicable.

5-iv) Quality assurance methods

Not applicable.

5-v) Ensure replicability by publishing the source code, and/or providing screenshots/screen-capture video, and/or providing flowcharts of the algorithms used

An example of CarePartner feedback content is included.

5-vi) Digital preservation

Not applicable.

5-vii) Access

The manuscript makes clear that this intervention was provided as part of a research study to VA patients.

5-viii) Mode of delivery, features/functionalities/components of the intervention and comparator, and the theoretical framework

Patients, CarePartners, and in-home caregivers (when present) randomized to standard mHealth were mailed information about HF self-care [24]. Patients received weekly IVR monitoring and self-management support calls for 12 months. Up to nine call attempts per week were made at times the patient indicated were convenient. IVR calls included recorded information and questions that patients answered using their touchtone keypad. The IVR calls were developed by a panel including primary care physicians, cardiologists, nurses, and experts in health behavior change and mHealth. Calls lasted roughly 10 minutes and followed a tree-structured algorithm to ask about overall health, HF symptoms, and self-management behaviors. Patients received pre-recorded information tailored to their reported symptoms and self-care practices.

When patients reported an urgent issue via IVR (i.e., worsening shortness of breath or weight increases), the system automatically issued a fax notification to their clinician. A significant weight increase was defined as a five pound increase over one or two weeks, a seven pound increase over three weeks, or an average gain of two pounds per week since the last automated call if more than three weeks had elapsed. Actions taken by clinicians based on the faxes were not tracked.

The mHealth+CP intervention was based on self-regulation theory, which emphasizes communication of expectations of behavior ("standards"), promotion of motivation to meet standards, and monitoring with feedback regarding the gap between behavior and standards [31, 32]. Patients and CarePartners randomized to mHealth+CP received identical intervention elements described above, plus CarePartners were automatically emailed a structured report after each completed IVR call. Reports described in lay language what patients' responses meant in terms of risk for HF exacerbations and included suggestions for how CarePartners could support self-management. CarePartners were asked to call their patient-partner weekly to review the reports and address identified issues.

CarePartners received guidelines about how to: communicate in a positive, motivating way; avoid conflict by respecting boundaries; include in-home caregivers; and respect confidentiality. Patients received a notebook including reminders and tips for their weekly patient-CarePartner calls. CarePartners received logbooks for tracking IVR reports, upcoming patient contacts, clinical encounters, and medication refills.

5-ix) Describe use parameters

See above.

5-x) Clarify the level of human involvement

See above.

5-xi) Report any prompts/reminders used

See above.

5-xii) Describe any co-interventions (incl. training/support)

See above.

6a) CONSORT: Completely defined pre-specified primary and secondary outcome measures, including how and when they were assessed

Baseline, 6-Month, and 12-Month Surveys. Patients' HF-specific quality of life, self-care, and patient-CarePartner communication were measured via quantitative telephone surveys. Baseline sociodemographic variables included patients' age, race, marital status, employment status, educational attainment, and income. Patients' baseline depressive symptoms were measured using the 10-item version of the CES-D [33]. CarePartners completed online surveys at each time point, but the current analyses include only baseline CarePartner characteristics relevant to the comparability of groups at randomization.

The primary outcome was HF-specific quality of life at 12 months, as measured by the Minnesota Living with Heart Failure Questionnaire (MLHFQ) [34]. HF self-care behaviors were measured using the Revised Heart Failure Self-Care Behavior Scale (HFSCB) [3]. A measure of HF medication adherence was created using the HFSCB adherence items with which patients reported how often they: "took [their] pills every day," "took [their] pills as the doctor prescribed, i.e., took all of the doses of [their] pills," "always refilled prescriptions for [their] pills on time," and "had a system to help tell [them] when to take [their] pills." The adherence measure based on these items was designed to identify patients reporting perfect adherence (i.e., a binary measure identifying patients reporting "always" engaging in all four behaviors). Binary indicators for perfect adherence tend to correct for inflated adherence reporting [35, 36].

To identify changes in patient-CarePartner communication, three relationship dimensions were measured at each timepoint: First, as an objective measure of communication intensity, patients were asked how often over the prior six months they communicated with their CarePartner by phone. Analyses examined patients' likelihood of reporting that they spoke at least twice per week. Second, the affective dimension of CarePartner support was measured using items based on prior studies of caregiving relationships [37, 38]. Patients were asked how often they experienced each of six negative emotions when talking with their CarePartner (sadness, loneliness, anger, tension, guilt, or frustration), and analyses examined patients' likelihood of reporting that they regularly experience one or more of these emotions. Third, to understand patients' perspective of the difficulty involved in CarePartner communication, analyses examined participants' likelihood of agreeing or strongly agreeing that it was "difficult to talk with [their] CarePartner about [their] illness."

Weekly IVR Adherence and Symptom Reports. Patients' IVR medication adherence and symptom reports were examined as potential indicators of differences across arms in intervention effectiveness, because short-term reporting intervals often provide information that is more reliable and less prone to bias than retrospective recall surveys [39-41]. Patients were considered adherent if they reported "always" taking their HF medication exactly as prescribed in the past week. Patients were classified as experiencing shortness of breath if they reported being bothered by shortness of breath "daily" or "several days" in the prior week. Patients were coded as having a significant weight gain if their reported weight met criteria described above. Finally, patients were coded as having positive self-reported health if they reported that their overall health was "excellent" or "very good."

6a-i) Online questionnaires: describe if they were validated for online use and apply CHERRIES items to describe how the questionnaires were designed/deployed

Not applicable.

6a-ii) Describe whether and how "use" (including intensity of use/dosage) was defined/measured/monitored

VR call completion rates were calculated using one record per week of attempted IVR calls, i.e., 52 call-weeks per patient minus weeks in which the patient was on vacation or hospitalized. Logistic models were used to predict patients' likelihood of completing each weekly call as a function of arm, baseline characteristics, and number of weeks since enrollment. Statistical tests for the analyses of call completion rates were adjusted for clustering of call-weeks within patients.

6a-iii) Describe whether, how, and when qualitative feedback from participants was obtained

No qualitative data were collected.

6b) CONSORT: Any changes to trial outcomes after the trial commenced, with reasons

No changes to the outcomes were made after the trial commenced.

7a) CONSORT: How sample size was determined

7a-i) Describe whether and how expected attrition was taken into account when calculating the sample size

The primary outcome was change in HF-specific quality of life between baseline and 12 months. The study was powered to detect a medium/small effect (D=.351) assuming a 20% loss to follow-up, similar to that observed in the prior HF trial by Sisk et al [42].

7b) CONSORT: When applicable, explanation of any interim analyses and stopping guidelines

Not applicable.

8a) CONSORT: Method used to generate the random allocation sequence

Pairs were randomized by a research associate within strata defined by whether the patient had an in-home caregiver. Sealed randomization envelopes were created by the study coordinator in blocks using an online random number generator. It was impossible to blind patients to their random assignment because patients and CarePartners were aware whether the CarePartner received email feedback.

8b) CONSORT: Type of randomisation; details of any restriction (such as blocking and block size)

Pairs were randomized within strata defined by whether the patient had an in-home caregiver. Sealed randomization envelopes were created by the study coordinator in blocks using an online random number generator.

9) CONSORT: Mechanism used to implement the random allocation sequence (such as sequentially numbered containers), describing any steps taken to conceal the sequence until interventions were assigned

See above.

10) CONSORT: Who generated the random allocation sequence, who enrolled participants, and who assigned participants to interventions

See above.

11a) CONSORT: Blinding - If done, who was blinded after assignment to interventions (for example, participants, care providers, those assessing outcomes) and how

11a-i) Specify who was blinded, and who wasn't

It was impossible to blind patients to their random assignment because patients and CarePartners were aware whether the CarePartner received email feedback.

11a-ii) Discuss e.g., whether participants knew which intervention was the "intervention of interest" and which one was the "comparator"

Patients were informed at the time of enrollment that there were two groups - one receiving CarePartner feedback and one that did not.

11b) CONSORT: If relevant, description of the similarity of interventions

Not applicable.

12a) CONSORT: Statistical methods used to compare groups for primary and secondary outcomes

Subsequent analyses compared patients and CarePartners across arms in the sample with outcome data. IVR call completion rates were calculated using one record per week of attempted IVR calls, i.e., 52 call-weeks per patient minus weeks in which the patient was on vacation or hospitalized. Logistic models were used to predict patients' likelihood of completing each weekly call as a function of arm, baseline characteristics, and number of weeks since enrollment. Statistical tests for the analyses of call completion rates were adjusted for clustering of call-weeks within patients.

The primary outcome was change in HF-specific quality of life between baseline and 12 months. The study was powered to detect a medium/small effect (D=.351) assuming a 20% loss to follow-up, similar to that observed in the prior HF trial by Sisk et al [42]. All outcomes were analyzed on an intent-to-treat basis. The xtmixed and logistic regression commands in Stata version 13.1 [43] were used to identify intervention effects on patients' HF-related quality of life, self-care, and patient-CarePartner communication. Predictors for each analysis included an indicator for arm, time (baseline, 6-month, and 12-month), and an arm-by-time interaction. Effect estimates presented in Table 2 represent differences across arms adjusted for baseline values. To examine differences across arms in IVR reports of medication adherence and symptoms, graphical displays were created illustrating the proportion of patients reporting a given outcome each week, separately by arm. Logistic regression models were fitted to predict patients' weekly IVR-reported outcomes, with weekly reports clustered within patient. Models included the following predictors: arm, time, and an arm-by-time interaction term. Variances for the estimated intervention effects were adjusted for the within-patient correlation of IVR reports across weeks [44-46]. To illustrate the magnitude of intervention-control differences in IVR-reported outcomes, the probability for each outcome at week 26 and 52 was predicted based on the logistic model separately for mHealth+CP and standard mHealth groups.

12a-i) Imputation techniques to deal with attrition / missing values

The sample included all patients with 12-month surveys plus 22 patients for whom 6-month survey data were carried forward. Initial analyses compared the baseline characteristics of patients who did versus did not have 12-month data in the imputed sample.

12b) CONSORT: Methods for additional analyses, such as subgroup analyses and adjusted analyses

Finally, a potential interaction was examined between patients' baseline level of depressive symptoms (CES-D) and arm, with respect to IVR reports of excellent/very good health. Specifically, patients' unadjusted frequency of reporting excellent/very good health was examined graphically as described above, within subgroups defined by baseline CES-D scores. Because graphical displays suggested an inflection point with two very different slopes, we fit logistic models separately for patients with CES-D scores that were low (0-4) versus high (5-10). Each model included terms for arm, baseline CES-D score, an arm-by-CES-D interaction, time, and an arm-by-time interaction.

RESULTS

13a) CONSORT: For each group, the numbers of participants who were randomly assigned, received intended treatment, and were analysed for the primary outcome

A total of 4,140 potentially eligible patients were identified from electronic medical records. Of these, 372 were randomized, and 331 (89%) had outcome data at 12-months (see CONSORT Figure 1). Patients lost to follow-up were less likely to report at baseline that they spoke with their CarePartner at least twice per week (43.9% versus 65.9%, p=.006) and had better baseline HF self-care scores as measured by the HFSCB (p=.002) but were not significantly different from patients with follow-up data on any other characteristic shown in Table 1 (see Electronic Supplementary Material 2 for details).

13b) CONSORT: For each group, losses and exclusions after randomisation, together with reasons

See CONSORT Figure 1.

13b-i) Attrition diagram

No diagram is included. The results state that:

Patients participated for a total of 15,709 call-weeks, during which they completed 14,175 calls, for a completion rate of 90.2%. IVR completion rates were essentially the same between mHealth+CP and standard mHealth arms (90.8% versus 89.7%), and there was no change in patients' likelihood of completing IVR calls throughout follow-up (p=.19). The likelihood of call completion was unrelated to patients' baseline HF-specific quality of life (MLHFQ) scores, HF self-management scores, CES-D scores, or measures of patient-CarePartner relationship quality (all p-values ≥ .15).

14a) CONSORT: Dates defining the periods of recruitment and follow-up

Patients were recruited from VA Cleveland Medical Center outpatient clinics between June 2009 and January 2012 and were followed for 12 months.

14a-i) Indicate if critical "secular events" fell into the study period

Not applicable.

14b) CONSORT: Why the trial ended or was stopped (early)

Not applicable.

15) CONSORT: A table showing baseline demographic and clinical characteristics for each group

See Table 1.

15-i) Report demographics associated with digital divide issues

See Table 1.

16a) CONSORT: For each group, number of participants (denominator) included in each analysis and whether the analysis was by original assigned groups

16-i) Report multiple "denominators" and provide definitions

See Table 1 and CONSORT figure 1

16-ii) Primary analysis should be intent-to-treat

The primary outcome was change in HF-specific quality of life between baseline and 12 months. The study was powered to detect a medium/small effect (D=.351) assuming a 20% loss to follow-up, similar to that observed in the prior HF trial by Sisk et al [42]. All outcomes were analyzed on an intent-to-treat basis.

17a) CONSORT: For each primary and secondary outcome, results for each group, and the estimated effect size and its precision (such as 95% confidence interval)

See Table 2 and the following text from the Legend for Figure 2 and Figure 3:

Figure 2. Unadjusted self-care and health status reports for patients in each randomization group by week since enrollment. Standard mHealth=patients randomized to IVR monitoring and self-care support with clinician alerts. mHealth+CP=patients randomized to the same intervention + weekly feedback to patients' CarePartners. The Y-axis for each panel differs in scale; bars represent the proportion of patients responding with that report. P-values are from logistic regression models testing differences across arms. P-values <.05 represent significant effects favoring mHealth+CP. A: Reports of always taking heart failure medication exactly as prescribed in the prior week. Main effect of arm ß =.5092 (95% CI: .0857, .9329). There was no significant arm-by-time interaction (p=.407) and the interaction term was removed from the final model. B: Reports of being bothered by shortness of breath every day or several days in the prior week. The main effect of arm was not statistically significant (.0894; 95% CI: -.2857, .4644; p=.640). Arm-by-time interaction ß=-.0114 (95% CI: -.0206, -.0022). C: Clinically significant weight gain generating a notification to patients' healthcare team. The main effect of arm was not statistically significant (ß=.0454; 95% CI: -.2147, .3055; p=.732). Arm-by-time interaction ß=-.0148 (95% CI: -.0232, -.0064). D: Reports of very good or excellent health (versus good, fair, or poor health) in the prior week. Main effect of arm, ß = -.1469 (95% CI: -.5366, -.2427). There was no significant arm-by-time interaction (p=.702) and the interaction term was removed from the final model.

Figure 3. Unadjusted reports of excellent/very good health for patients in each randomization group by baseline CES-D depression score. Solid bars represent standard mHealth patients. White bars represent mHealth+CP patients. Among patients with CES-D scores 0-4, the main effect for arm was ß =-.46, CI: -.90, .028; p=.037, with no significant arm-by-CES-D interaction. Among patients with CES-D scores 5+, the main effect for arm was ß =1.27, CI: .42, 2.12; p=.003, with no significant arm-by-time interaction.

17a-i) Presentation of process outcomes such as metrics of use and intensity of use

Patients participated for a total of 15,709 call-weeks, during which they completed 14,175 calls, for a completion rate of 90.2%. IVR completion rates were essentially the same between mHealth+CP and standard mHealth arms (90.8% versus 89.7%), and there was no change in patients' likelihood of completing IVR calls throughout follow-up (p=.19). The likelihood of call completion was unrelated to patients' baseline HF-specific quality of life (MLHFQ) scores, HF self-management scores, CES-D scores, or measures of patient-CarePartner relationship quality (all p-values ≥ .15). IVR calls generated fax notifications to clinicians 1,606 times (11.3% of completed calls), including 743 for weight gain, 774 for shortness of breath, and 89 for both.

17b) CONSORT: For binary outcomes, presentation of both absolute and relative effect sizes is recommended

See Table 2 and Figures 2-3.

18) CONSORT: Results of any other analyses performed, including subgroup analyses and adjusted analyses, distinguishing pre-specified from exploratory

See Figure 3 and the following text from the results.

The strong negative association seen among standard mHealth patients between baseline CES-D scores and IVR reports of excellent/very good health was not apparent in the mHealth+CP arm (Figure 3). The leveling of mHealth+CP patients' perceived health reports across baseline CESD-levels reflected a somewhat lower proportion reporting excellent/very good health relative to standard mHealth patients when baseline CES-D scores were low, as well as a substantially higher proportion reporting excellent/very good health among those with greater depressive symptoms. In both the low CES-D (scores 0-4) and high CES-D (5+) strata, the coefficient for the main effect of arm was significant, although in opposite directions, with no arm-by-CES-D interaction within strata. The positive intervention effect among patients with higher baseline CES-D scores (ß =1.27, p=.003) was substantially greater than the magnitude of the negative intervention effect among patients with lower scores (ß =-.46, p=.037). According to these models, patients with a baseline CES-D score of 1 were 11% less likely to report excellent/very good health if randomized to mHealth+CP, while patients with a baseline CES-D score of 8 were 22% more likely if randomized to mHealth+CP relative to usual care.

18-i) Subgroup analysis of comparing only users

Not applicable.

19) CONSORT: All important harms or unintended effects in each group

We believe that there were no important harms or unintended effects.

19-i) Include privacy breaches, technical problems

Not applicable.

19-ii) Include qualitative feedback from participants or observations from staff/researchers

Qualitative feedback was not collected.

DISCUSSION

20) CONSORT: Trial limitations, addressing sources of potential bias, imprecision, multiplicity of analyses

20-i) Typical limitations in ehealth trials

This trial had several limitations. Prior studies have shown that patients' medication self-reports are highly correlated with objective measures of medication use, especially when the recall interval is short and the measure is designed to identify even mild forms of non-adherence [35, 36]. However, it would be important to confirm these findings with medication refill data. Similarly, it would be useful to verify patients' self-reported weights using data-storing electronic scales. Another limitation is that the trial was conducted among VA patients, nearly all of whom were men. Caregiving dynamics differ by patients' demographic and clinical characteristics, and future studies should determine whether results can be replicated in other populations, including non-VA patients and women. Some important clinical information about participants was not collected during the trial. For example we do not know whether patients underwent cardiac surgery, resynchronization therapy, or revascularization. While we have no indication that randomization was unsuccessful, and patients in both groups were well matched on a wide range of baseline characteristics, it remains possible that unobserved differences in patients' clinical status at the time of enrollment may have contributed to the intervention effects observed. Finally, our study had several outcomes measured at two time points, and multiple comparisons may have contributed to the findings. However, results were consistent with the study's theoretical framework, and significant results were consistently in the same direction, i.e., favoring mHealth+CP over standard mHealth.

21) CONSORT: Generalisability (external validity, applicability) of the trial findings

21-i) Generalizability to other populations

See limitations section, above.

21-ii) Discuss if there were elements in the RCT that would be different in a routine application setting

See limitations section, above.

22) CONSORT: Interpretation consistent with results, balancing benefits and harms, and considering other relevant evidence

22-i) Restate study questions and summarize the answers suggested by the data, starting with primary outcomes and process outcomes (use)

In this randomized comparative effectiveness trial, no group differences were identified at 6 or 12 months in the primary outcome of HF-specific quality of life or the composite measure of HF self-care. However, a number of potentially important differences in the process and outcomes of care were identified that favored mHealth+CP. For example, in both follow-up surveys, a greater proportion of mHealth+CP patients reported perfect medication adherence, and mHealth+CP patients were consistently more likely throughout the one-year follow-up to report via IVR that they took their HF medications as prescribed during the prior week. mHealth+CP patients also had a significantly greater decrease in their likelihood of reporting shortness of breath via IVR and were less likely to report clinically-significant weight gains.

22-ii) Highlight unanswered new questions, suggest future research

In this randomized comparative effectiveness trial, no group differences were identified at 6 or 12 months in the primary outcome of HF-specific quality of life or the composite measure of HF self-care. However, a number of potentially important differences in the process and outcomes of care were identified that favored mHealth+CP. For example, in both follow-up surveys, a greater proportion of mHealth+CP patients reported perfect medication adherence, and mHealth+CP patients were consistently more likely throughout the one-year follow-up to report via IVR that they took their HF medications as prescribed during the prior week. mHealth+CP patients also had a significantly greater decrease in their likelihood of reporting shortness of breath via IVR and were less likely to report clinically-significant weight gains.

Other information

23) CONSORT: Registration number and name of trial registry

The trial was registered in ClinicalTrials.gov (#NCT00555360).

24) CONSORT: Where the full trial protocol can be accessed, if available

We note that the trial was registered in ClinicalTrials.gov (#NCT00555360) and the protocol can be accessed there..

25) CONSORT: Sources of funding and other support (such as supply of drugs), role of funders

Sources of funding have been noted.

X26-i) Comment on ethics committee approval

The study protocol was approved by the Ann Arbor VA Human Subjects Committee, and all patients provided written informed consent. None of the authors had any financial conflict of interest.

x26-ii) Outline informed consent procedures

Eligible and interested patients were mailed informed consent forms. CarePartners provided verbal consent to participate.

X26-iii) Safety and security procedures
Not applicable.
X27-i) State the relation of the study team towards the system being evaluated
We note that we had no conflict of interest.
