# Supplementary material for: A Mobile Health Intervention Supporting Heart Failure Patients and Their Informal Caregivers: A Randomized Comparative Effectiveness Trial
Source: J Med Internet Res. 2015 Jun 10;17(6):e142. doi: 10.2196/jmir.4550 (PMC4526929; doi:10.2196/jmir.4550)
Supplement: Multimedia Appendix 5 [file jmir_v17i6e142_app5.pdf]

**Multimedia Appendix 5. Comparison of Baseline Characteristics for Patients with and without Follow-up Data**

| | All Enrollees N=372 | Analytic Sample n=331 | Lost to Follow-up n=41 | p-value |
|---|---|---|---|---|
| **Patient Characteristics** | | | | |
| Age in years | 67.9 ± 10.2 | 67.8 ± 10.2 | 68.4 ± 10.6 | .722 |
| Male | 98.9 | 99.4 | 95.1 | .012 |
| White race | 76.6 | 77.0 | 73.2 | .830 |
| Married/Partnered | 58.9 | 58.9 | 58.5 | .963 |
| High School or less | 49.2 | 48.0 | 58.5 | .211 |
| Live alone | 32.8 | 32.6 | 34.1 | .845 |
| Unemployed/retire | 87.9 | 87.6 | 90.2 | .626 |
| Income < $15,000 | 31.7 | 31.4 | 34.1 | .772 |
| CES-D Depression | 3.0 ± 2.5 | 2.97 ± 2.49 | 3.56 ± 2.05 | .143 |
| MLHFQ[a] | 44.2 ± 25.6 | 43.3 ± 25.3 | 50.7 ± 27.1 | .096 |
| HFSCB[b] | 83.8 ± 18.1 | 82.8 ± 17.9 | 91.8 ± 17.9 | .002 |
| Adherent to HF Rx[c] | 52.4 | 52.3 | 53.7 | .866 |
| **Relationship Quality[d]** | | | | |
| Talk 2+ times/ week | 63.4 | 65.9 | 43.9 | .006 |
| Negative emotions[e] | 45.8 | 44.8 | 53.7 | .284 |
| Perceived difficulty[f] | 21.5 | 21.5 | 22.0 | .941 |

**Multimedia Appendix 5 (continued). Comparison of Baseline Characteristics for Patients with and without Follow-up Data**

| | All Enrollees N=372 | Analytic Sample n=331 | Lost to Follow-up n=41 | p-value |
|---|---|---|---|---|
| **CarePartner Characteristics** | | | | |
| Age | 47.1 ± 13.2 | 46.7 ± 13.2 | 49.7 ± 12.5 | .171 |
| Male | 34.4 | 35.0 | 29.3 | .424 |
| Married/Partnered | 67.7 | 68.6 | 61.0 | .358 |
| High School or less | 27.7 | 27.8 | 26.8 | .924 |
| Unemployed/retired | 37.1 | 36.9 | 39.0 | .762 |

Notes: Cell entries are either mean ± SD or percents. [a]Minnesota Living with Heart Failure Questionnaire Scores. Lower scores indicate better functioning. [b]Revised Heart Failure Self-Care Behavior Scale. Higher scores indicate better HF self-care. [c]Percent of patients with perfect HF medication adherence over the prior month as measured by the four HFSCB items focused on adherence (see Methods). [d]Patients' reports regarding their relationship with their CarePartner. [e]Percent of patients who report regularly experiencing any of six negative emotions when talking with their CarePartner (sadness, loneliness, anger, tension, guilt, or frustration). [f]Percent of patients who agree that it is "difficult to talk to [their] CarePartner about [their] illness.
